# Design and Investigation of Superatoms for Redox Applications: First-Principles Studies

**DOI:** 10.3390/mi15010078

**Published:** 2023-12-29

**Authors:** Celina Sikorska

**Affiliations:** 1 Faculty of Chemistry, University of Gdańsk, Fahrenheit Union of Universities in Gdańsk, Wita Stwosza 63, 80-308 Gdańsk, Poland; celina.sikorska@ug.edu.pl; 2 Department of Physics, The University of Auckland, Private Bag 92019, Auckland 1142, New Zealand

**Keywords:** superatoms, cluster-assembled materials, semiconductors, computational chemistry, anions, cations, desirable materials, perovskites, Li-ion batteries, carbon dioxide conversion

## Abstract

A superatom is a cluster of atoms that acts like a single atom. Two main groups of superatoms are superalkalis and superhalogens, which mimic the chemistry of alkali and halogen atoms, respectively. The ionization energies of superalkalis are smaller than those of alkalis (<3.89 eV for cesium atom), and the electron affinities of superhalogens are larger than that of halogens (>3.61 eV for chlorine atom). Exploring new superalkali/superhalogen aims to provide reliable data and predictions of the use of such compounds as redox agents in the reduction/oxidation of counterpart systems, as well as the role they can play more generally in materials science. The low ionization energies of superalkalis make them candidates for catalysts for CO_2_ conversion into renewable fuels and value-added chemicals. The large electron affinity of superhalogens makes them strong oxidizing agents for bonding and removing toxic molecules from the environment. By using the superatoms as building blocks of cluster-assembled materials, we can achieve the functional features of atom-based materials (like conductivity or catalytic potential) while having more flexibility to achieve higher performance. This feature paper covers the issues of designing such compounds and demonstrates how modifications of the superatoms (superhalogens and superalkalis) allow for the tuning of the electronic structure and might be used to create unique functional materials. The designed superatoms can form stable perovskites for solar cells, electrolytes for Li-ion batteries of electric vehicles, superatomic solids, and semiconducting materials. The designed superatoms and their redox potential evaluation could help experimentalists create new materials for use in fields such as energy storage and climate change.

## 1. Introduction

A superatom is a cluster of atoms that acts like a single atom [1,2,3]. Its features can be precisely controlled by adding, substituting, or removing a single atom within it. Two main groups of superatoms are superalkalis and superhalogens, which mimic the chemistry of alkali and halogen atoms, respectively. Typical representatives of superalkalis are FLi_2_ [4], OLi_3_ [5], and SiLi_5_ [6], while superhalogens are LiF_2_ [7], MgCl_3_ [8], AlCl_4_ [9]. A superalkali species has one electron more, while a superhalogen species has one electron less than needed for octet shell closure. The ionization energies (IEs) of superalkalis are smaller than those of alkalis (<3.89 eV for cesium atom), and the electron affinities (EAs) of superhalogens are larger than that of halogens (>3.61 eV for chlorine atom). Therefore, superalkalis can act as reducing agents [10,11] and superhalogens are oxidizing agents [12,13].

The existence of superhalogens was predicted theoretically in 1981 by Gutsev and Boldyrev, who proposed a simple formula describing one class of these compounds, MX_k+1_ [14]. According to the MX_k+1_ formula, a superhalogen is a neutral system containing a k-valent central atom M (a main group or transition metal atom) decorated with k + 1 halogen atoms (where k is the maximal formal valence of the atom M) and can form a stable anionic system having large additional electron binding energy. Since the 1980s, many theoretical efforts have been made to estimate the vertical electron detachment energies (VDEs) of various anions having superhalogens as their neutral parents, such as BF_4_^−^, AlCl_4_^−^, SiCl_5_^−^, or AsF_6_^−^ [7,9,14,15,16,17,18,19,20,21]. The first experimental photoelectron spectra of superhalogens were measured for MX_2_^−^ anions (where M = Li, Na, and X = Cl, Br, I) by Wang’s group in 1999 [16]. This experimental confirmation of the existence of superhalogen became a milestone and resulted in bringing more attention to this class of chemical species. Examples include studies on transition metal superhalogen anions MCl_4_^−^ (M = Sc, Y, La) [22], the oxygen-based BO_2_^−^ [23], and even larger species such as [Na_x_Cl_x+1_]^−^ (n = 1–4) [24].

Reducing agents play an essential role in chemical synthesis. Strong reductors have low ionization energies. Among the periodic table elements, alkali metal atoms exhibit the lowest ionization energies (3.89–5.39 eV). Superalkalis have even lower ionization energies than those of the alkali metal atoms. In 1982, Gutsev and Boldyrev introduced a simple ML_k+1_ formula to describe one class of superalkalis, where M is a k-valent electronegative central atom ligated with k + 1 alkali-metal atoms (L) [25]. The ML_k+1_ molecular system tends to lose one valence electron, forming a very stable cation with the positive charges distributed over all the k + 1 alkali atoms (L). The existence of OL_3_ (L = Li, Na, K), ML_2_ (M = F, Cl, Br, I; L = Li, Na, K), SLi_3_, Li_3_F_2_, Li_2_CN, and Na_2_CN superalkalis have been confirmed by experiments [26]. Since the early 1980s, the scope of superalkalis has been expanded through the introduction of binuclear superalkali species (M_2_L_2k+1_) [27,28] and polynuclear superalkalis (YL_k+1_, where k is the valence state of the anionic groups/clusters) with various polyatomic groups (Y) [29,30]) as the central cores. More recently, substantial effort has been devoted to proposing alternative superalkali species, including non-metallic superalkali cations [31], aromatic superalkali species [32], organo-zintl superalkalis [33], and superalkalis with boron atom acting as a central atom [34]. Despite these achievements, it is still desirable to obtain novel superalkali species to seek even lower ionization energies.

Exploring new superalkali/superhalogen aims to provide reliable data and predictions of the use of such compounds as redox agents in the reduction/oxidation of counterpart systems, as well as the role they can play more generally in materials science. The low ionization energies of superalkalis make them candidates for catalysts for CO_2_ conversion into renewable fuels and value-added chemicals. The large electron affinity of superhalogens makes them strong oxidizing agents for bonding and removing toxic molecules from the environment. By using the superatoms as building blocks of cluster-assembled materials, we can achieve the functional features of atom-based materials (like conductivity or catalytic potential) while having more flexibility to achieve higher performance. Superatoms could be substituted for atoms as functional units because they potentially possess atomic-like functions (including redox activity). Furthermore, there are fewer than a hundred naturally stable elements, whereas the variety of superatoms is almost endless. Successful design of superatom by means of molecular modeling will allow experimentalists to create new materials for use in fields such as energy and climate change.

The results discussed in the present paper can be applied to create a new class of desirable materials for applications in sustainable energy generation, nanoelectronics, and energy storage. The designed superatoms enrich a library of molecular clusters that can act as building blocks of self-assembled materials. The self-assembled materials offer a unique approach to creating complex yet adjustable atomically precise materials. The main application areas of superatoms include 2D materials, ferroelectrics, energy storage, CO_2_ activation, and its conversion into fuels. This feature paper demonstrates how structure modifications of the superatoms allow for the tuning of the electronic structure and might be used to create unique functional materials. Based on my first-principles calculations, I demonstrated that superatoms can form stable perovskites for optoelectronic devices such as solar cells, electrolytes for Li-ion batteries of electric vehicles, superatomic solids, and semiconducting materials.

## 2. Scope and Objectives of Research

The scope of the research discussed in this paper encompasses chemical compounds with extreme physicochemical features related to the electron attachment and detachment processes—high electron affinities (EA) and low ionization energies (IE), respectively. The studies cover the issues of designing such compounds, accurately estimating the additional electron binding energy and ionization energy, as well as evaluating their application potential. All problems were solved using the ab initio computational methods of quantum chemistry.

Even though superatomic systems (superalkalis and superhalogens) have continued to receive a lot of attention in chemistry since the 1980s [25] and have recently been used in materials chemistry [35], there are still some issues, hitherto unidentified in the literature concerning these chemical compounds that mimic elemental atoms. Among the most critical challenges related to the design of new chemical substances with useful, in terms of application, physical and chemical properties are those related to the stability (geometric, thermodynamic, and electronic) of the designed molecules, as well as the complexity of the relationship between electronic structure and physicochemical properties. In the case of polyatomic molecular systems, the large number of atoms implies difficulties in conducting a reliable stability assessment based on highly correlated quantum-mechanical calculations. The strong motivation to perform extensive quantum-mechanical calculations for designing new superatoms is their ability to create new materials that will be cheaper and more effective in chemical processes. For example, palladium, a jet engine catalyst, costs $100 per gram. Zirconium oxide, which can replace it, costs $0.02 per gram. Consequently, zirconium oxide, being isoelectronic with palladium, is 5000 times cheaper than palladium. Thus, the design of chemical compounds that mimic the properties of other elements creates the possibility of designing new materials with properties desirable from an industrial point of view.

**Figure 1:**
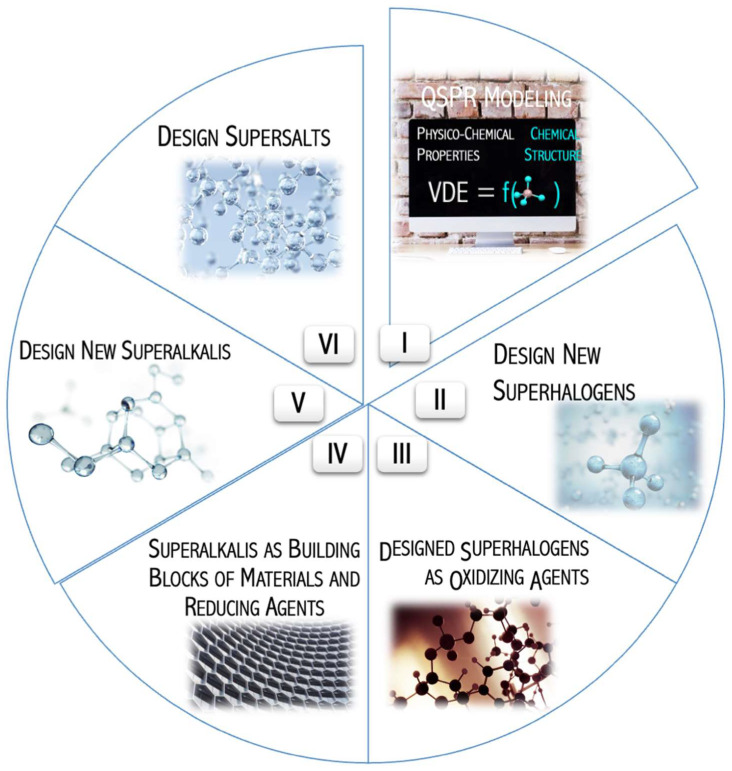
Scope of studies.

The above challenges and the unique characteristics of superatoms mimicking alkali metals (superalkalis) and halogens (superhalogens) allowed me to identify six key research areas reflecting the goals and scope of recently conducted studies (Figure 1). They were:

- Develop a methodology for predicting the electron affinity of superhalogens using quantitative modeling of the structure-electronic stability relationship of superhalogen anions.
- Design new alternative superhalogens with large electron affinity.
- Examine the applicability of the designed superhalogens as oxidizers in chemical reactions.
- Assess the potential applications of superalkalis in materials and as reducing agents.
- Design compounds with low ionization potentials (superalkalis).
- Verify the ability to form stable crystal structures built of superalkali cations and superhalogen anions.

It is noteworthy that the above thematic areas are interpenetrate and even complement each other, thus offering the possibility of a comprehensive assessment of the electron-withdrawing properties of superatoms and the sustainable design of new functional materials composed of superatoms instead of expensive or inaccessible chemical elements (such as precious metals).

## 3. Discussion of Selected Recent Studies on Superatomic Systems

This feature paper describes the first-principles studies on designing polyatomic molecular systems with strong electron-acceptor (superhalogens) or electron-donor (superalkalis) properties and potential chemical applications in material chemistry. These research studies not only describe novel superatoms but also verify the usefulness of computational chemistry and solid-state physics methods to predict the stability and physicochemical properties of chemical compounds, supervised learning methods for classification and prediction with a particular focus on the use of these methods in assessing the application potential of superatoms. A concise description of selected research projects is presented in the following sections.

### 3.1. Prediction of the Electronic Stability of Superhalogen Anion from Topology

**Figure 2:**
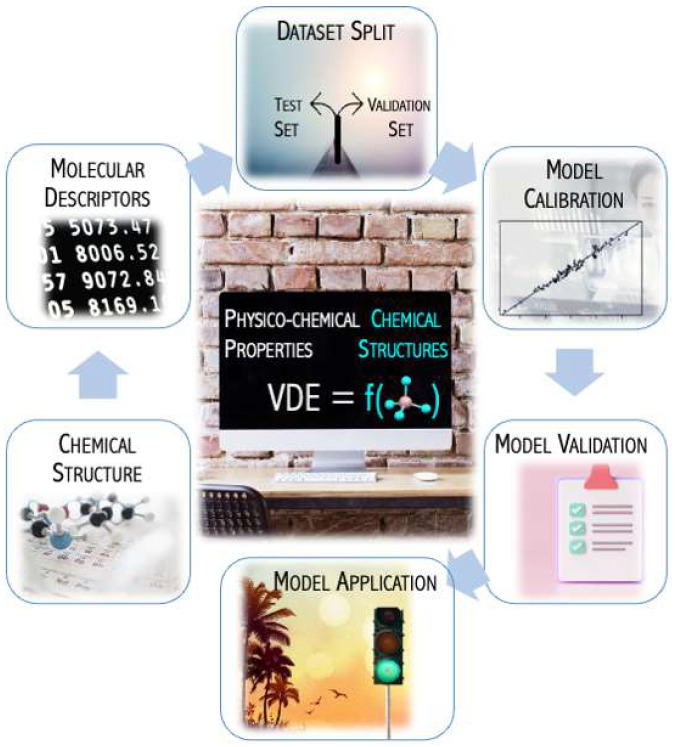
The idea of QSPR modeling.

Negatively charged molecular systems play an important role in chemical reactions (both as the reagents and reaction products). Due to their potential applications in material chemistry, anions are the subject of extensive theoretical and experimental studies. One class of such compounds is superhalogen anions, whose large electronic stabilities may lead to the forming of unusual chemical materials. An unusual number of ligands in relation to the valency of the central atom has a stabilization effect on the ability of the superhalogen systems to bind an extra electron. To investigate the relationship between superhalogen’s properties of electron affinity and their structure and composition, the quantitative structure-property relationship (QSPR) approach can be used. The QSPR technique mathematically links physicochemical properties with the structure of a molecule (Figure 2). Descriptors are the quantitates that represent attributes of the molecules and aid in establishing the mathematical correlation. The usefulness of this method has been confirmed for different compounds (such as nanoparticles) [35].

In [36], the performance of the QSPR approach was tested to suggest an effective method that could be used to predict the electronic stability of superhalogen anions. A QSPR model was developed to relate superhalogen structural features with vertical electron detachment energy (VDE) for a set of MX_4_^−^ (M = B, Al; X = F, Cl, Br) anions. The methodology applied is schematically presented in Figure 2 and involves the following steps: (i) collecting data set; (ii) calculating molecular descriptors for all studied compounds; (iii) splitting the compounds into training and validation sets; (iv) calibrating a QSPR model, (v) internal and external validating the model with the use of a test and validation sets, respectively.

The Genetic Algorithm was applied, combined with the Multiple Linear Regression approach to select variables and develop models. Relying on the evolutionary operations of ‘crossover and mutation’, the optimal combinations of descriptors that explain property variation within the training set were chosen. The most significant QSPR model is provided below (Equation (1) [36]):

VDE = 4.875(±0.101) + 4.287(±0.166) · SpMax4_Bh(s) + 1.708(±0.157) · ATS1m(1)

where N = 20, R^2^ = 0.975, Q^2^_CV_ = 0.968, Q^2^_EXT_ = 0.960, RMSE_C_ = 0.146, RMSE_CV_ = 0.167, RMSE_EXT_ = 0.190.

**Figure 3:**
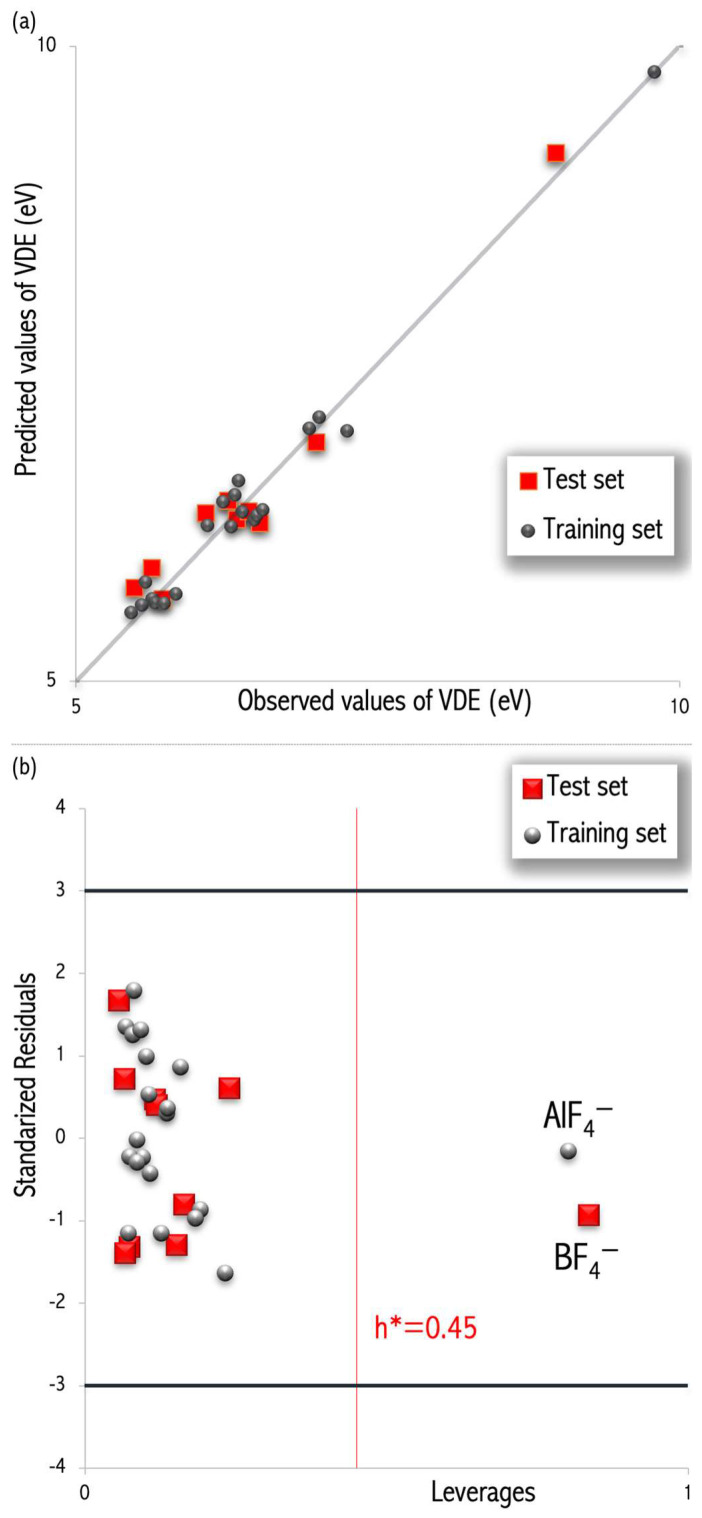
(**a**) The plot of observed vs. predicted VDEs (in eV), (**b**) the Williams plot of the developed QSPR model. The red vertical line corresponds to the leverage threshold (h*). Adapted with permission from ref. [36]. Copyright 2015 Elsevier B.V.

The descriptors (SpMax4_Bh(s), ATS1m) depend on topology and will be discussed later (Equations (2)–(4)). The values of the determination coefficient (R^2^), the cross-validation determination coefficient (Q^2^_CV_), and the external validation coefficient (Q^2^_EXT_) are close to 1, while the corresponding root mean square error values (RMSE_C_, RMSE_CV,_ and RMSE_EXT_) are both low and similar. Hence, the developed QSPR model is well-fitted, robust, and has good predictive abilities. In addition, the visual correlation between the observed and the predicted VDE for the training and validation sets is also satisfactory (Figure 3a).

The leverage approach [37] was performed to verify the chemical applicability domain of the developed model [36]. The plot of the standardized residuals versus the leverage values (the Williams plot, Figure 3b) confirmed that most molecules from the calibration and validation sets are located inside a rectangular area within the leverage threshold h* = 0.45 and ±3 standard deviation units [36]. In the Williams plot, one compound (AlF_4_^−^) was found to be an X outlier characterized by a leverage threshold (red line in Figure 3b), and it can be explained as a compound with peculiar features poorly represented in the training set. However, this X-outlier has a small residual, and hence, it can be regarded as a stabilizing point, increasing the model’s precision [38]. Explicitly, if the h_i_ values of training compounds are higher than h* and, simultaneously, the predictions for those molecules have small residuals, such molecules are so-called ‘good influence points’, stabilizing the model and making it more precise.

The main advantage of the developed QSPR model is that both descriptors belong to topology molecular descriptors. The topology molecular descriptors are defined as quantifiable properties that can be estimated from the connection table representation of a molecule (e.g., elements, formal charges, and bonds) and do not require knowledge of the atomic coordinates. The topology descriptors are not dependent on the conformation of a molecule and are suitable for non-optimized chemical structure studies. The SpMax4_Bh descriptor is derived from the molecular graph and calculated from the Burden matrices, *Bh*(*I*). The Burden matrices are augmented adjacency matrices defined to account for heteroatoms and bond multiplicity as the following (Equation (2)):

(2) $${\left[Bh(I)\right]}_{ij}=\left\{\begin{array}{c} \sqrt{\pi_{ij}^{*}}\;if\;(i,j)\in E(G)\\ I_{i}\;if\;i=j\\ 0.001\;if\;\left(i,j\right)\notin E(G) \end{array}\right.$$

The diagonal elements are the intrinsic state (I-state) of atoms, while the off-diagonal elements (corresponding to pairs of bonded atoms) are the square roots of conventional bond orders π* (i.e., 1 for single bonds), and all other matrix elements are set at 0.001 [39]. The SpMax4_Bh(s) descriptor provides the fourth largest positive eigenvalue for each matrix weighted by intrinsic state (I-state). The intrinsic state (I-state) of the *i*th atom was estimated with Equation (3):

(3) $$I_{i}=\frac{{\left(\frac{2}{n_{i}}\right)}^{2}\delta_{i}^{\upsilon }+1}{\delta_{i}}$$

where *n_i_* is the principal quantum number of the last electronic shell, $\delta_{i}^{\upsilon }$ is the number of valence electrons, and $\delta_{i}$ is the number of sigma electrons of the ith atom in the molecular structure.

The SpMax4_Bh(s) descriptor strongly depends on the ligands which the superhalogen system consists of. The primary factor differentiating the intrinsic state values among halogen atoms is the principal quantum number of the last electronic shell (*n_i_*, Equation (3)). In fact, there is a strong relationship between the VDE value and the nature of the orbitals involved in the electron addition. The principal quantum number defines the energy of an electron and the size of the orbital (i.e., the distance from the nucleus of the peak in a radial probability distribution plot). As the principal quantum number increases, the orbital becomes larger, and the electron density is farther from the nucleus. As *n_i_* increases, the electron also has a higher potential energy and is, therefore, less tightly bound to the nucleus. Consequently, involving ligands with larger principal quantum numbers results in a VDE decrease.

The second descriptor in the developed QSPR model is the ATS (autocorrelation of topological structure) descriptor. The ATS descriptor is a measure of the probability of finding objects of defined properties within a distance of interest [40]. The distance between atoms is estimated as the number of bonds between the respective atoms (topological distance, i.e., the length (number of involved bonds) of the shortest path between the two atoms). The ATS1m descriptor is weighted by the atomic mass of atom pairs at a topological distance equal to 1. In other words, the ATS1m value is obtained by summing all the products *m_i_* × *m_j_* of all the pairs of atoms *i* and *j*, for which topological distance equals one as:

(4) $$ATS1m=\sum_{i=1}^{nAT-1}\sum_{j=i+1}^{nAT}m_{i}m_{j}\delta_{ij}$$

where *nAT* is the total number of molecule atoms and $\delta_{ij}$ is equal to one if atoms *i* and *j* are connected via chemical bond, zero otherwise.

The ATS1m descriptor (Equation (4)) depends on atomic mass. Different central atoms and ligands affect the VDE of the superhalogen anion. The most striking difference I observed while comparing the BX_4_^−^ and AlX_4_^−^, as the replacement of B central atom with Al leads to the electron binding energy (VDE) increase from 6.218 eV (BCl_4_^−^) to 7.016 eV (AlCl_4_^−^) [9]. The same pattern I found for BF_3_Cl^−^ (VDE = 6.428 eV) and AlF_3_Cl^−^ (VDE = 7.245 eV) as well as for the BF_2_Cl_2_^−^ (VDE = 6.089 eV) and AlF_2_Cl_2_^−^ (VDE = 6.990 eV) anions [9]. In the case of each BX_4_^−^/AlX_4_^−^ pair considered (X = F, Cl, Br), the increase in the VDE is accompanied by the increase of the atomic mass of the central atom. Namely, the boron atom (atomic mass equal to 9 a.u.) as a central atom provides a considerably smaller VDE value than those obtained for the corresponding species in which the role of the central atom plays aluminum (atomic mass equal to 27 a.u.). This observation is consistent with the changes in the ATS1m that can be derived from (Table 1 of [36]). As far as the influence of the ligands on the VDE values is concerned, in the BF_4_^−^/BCl_4_^−^/BBr_4_^−^ and AlF_4_^−^/AlCl_4_^−^/AlBr_4_^−^ series, the VDE decreases from 8.975 eV to 5.625 eV and from 9.789 eV to 6.346 eV, respectively [9]. This significant decrease in the VDE value caused by replacing four F ligands with either four Cl or four Br atoms shows that a larger atomic mass of the ligand results in a smaller VDE of the resulting superhalogen anion.

The interpretation of the physical meaning of the topological descriptors used in the developed QSPR model suggests that the electron stability of the MX_4_^−^ anions (M = B, Al; X = F, Cl, Br) can be predicted from the atomic mass of the atoms comprising the superhalogen systems. The highest VDE values are expected for species containing, at the same time, a central atom with a large atomic mass and ligands characterized by a small atomic mass. Indeed, the highest VDE is observed for the AlF_4_^−^ (VDE = 9.789 eV) anion and the replacement of one fluorine atom with Cl or Br leading to AlF_3_Cl^−^ (VDE = 7.245 eV) or AlF_3_Br^−^ (VDE = 6.519 eV) reduces the VDE by 2.544 and 3.270 eV, respectively [9]. This observation indicates that replacing only one small ligand in the superhalogen system with a larger atom is highly unfavorable because it reduces electronic stability. This issue will be discussed further in Section 3.4.

The developed QSPR model predicts VDE directly from the topology molecular graphs and conforms with the general predictions formulated previously for the superhalogen anions [9,14]. Explicitly, the observation that a smaller ‘size’ of the halogen ligands usually leads to a larger vertical electron detachment energy is confirmed by the developed model. Based on the analysis of the obtained results [36], it was demonstrated that the value of the VDE is closely related to the nature of the orbitals involved in electron addition, being the highest one within MF_4_^−^ (M = B, Al) and lower for ligands with higher principal quantum numbers. Moreover, the atomic mass of the central atom (B or Al in our case) and the atomic mass of ligands are strongly related to the VDE of the resulting anion.

**Figure 4:**
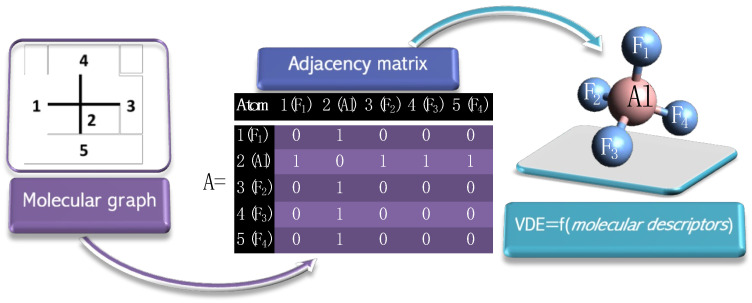
VDE prediction from topology molecular graph. Adapted with permission from ref. [36]. Copyright 2015 Elsevier B.V.

In paper [36], based on the two theoretical molecular descriptors calculated exclusively from the molecular structures, a QSPR model to estimate vertical electron detachment energies of MX_4_^−^ (M = B, Al; X = F, Cl, Br) superhalogen anions was developed. Both descriptors, as topology descriptors, do not depend on the conformation of a molecule and only use the atoms and connection information of the molecule for the calculation. The developed QSPR model, therefore, allows us to predict the electron binding energy of superhalogen anion based on the connection table representation of a molecule (Figure 4). The advantage of this approach lies in the fact that it requires only the knowledge of the topology and does not require experimental quantities or quantum-mechanical computations. Hence, the developed QSPR model could provide reliable VDE values of superhalogen anions in the absence of theoretical characterization (e.g., due to insufficient computer resources). Moreover, the QSPR model reveals the mass of atoms contributing to the superhalogen anions and the number of principal quantum number (*n_i_*) of ligands to be the most influential atomic properties in the structures of the superhalogen anions. The developed model and its interpretation described in detail in [36] might be useful for theoretical and experimental chemists, especially those who design new materials with strong electron-acceptor properties.

### 3.2. Design of Superhalogen Anions Containing of Noble Gas Atoms

**Figure 5:**
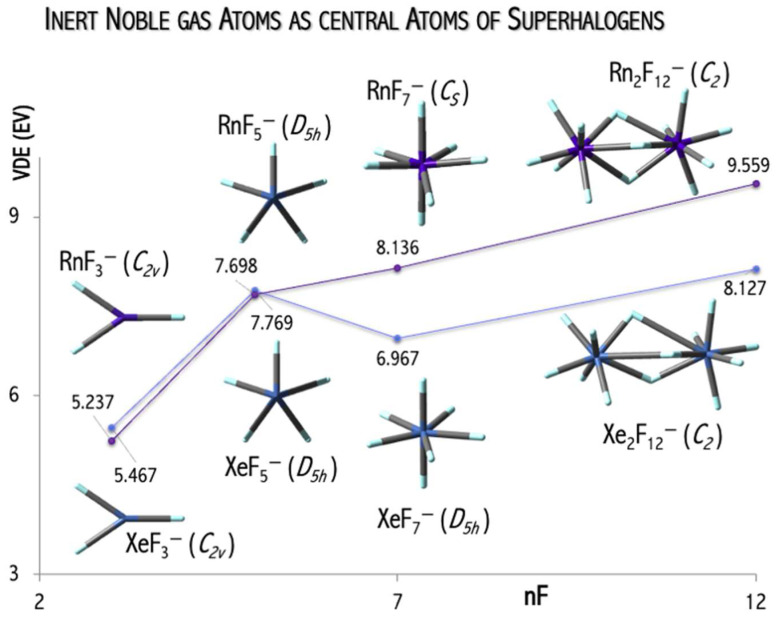
The vertical electron detachment energy (VDE in eV) of Xe_n_F_6n+1_^−^ (blue circles) and Rn_n_F_6n+1_^−^ (purple circles) anions vs. number of fluorine atoms (nF) as obtained from OVGF-6-311++G(3df,3pd)+ECPs level. The plot is based on data from ref. [43].

I designed and investigated theoretically superhalogen anions in which inert noble gas atoms play the role of central atoms. The previous theoretical studies [8,9,17,41,42] and developed QSPR model [36] allow us to assume that employing fluorine atoms could result in large electronic stability of the designed systems. Indeed, an ab initio calculation confirmed that mononuclear NgF_n_^−^ (n = 3, 5, 7) and binuclear Ng_2_F_13_^−^ anions (Ng = Xe, Rn; Figure 5) are geometrically, thermodynamically, and electronically stable systems in the gas phase [43].

**Figure 6:**
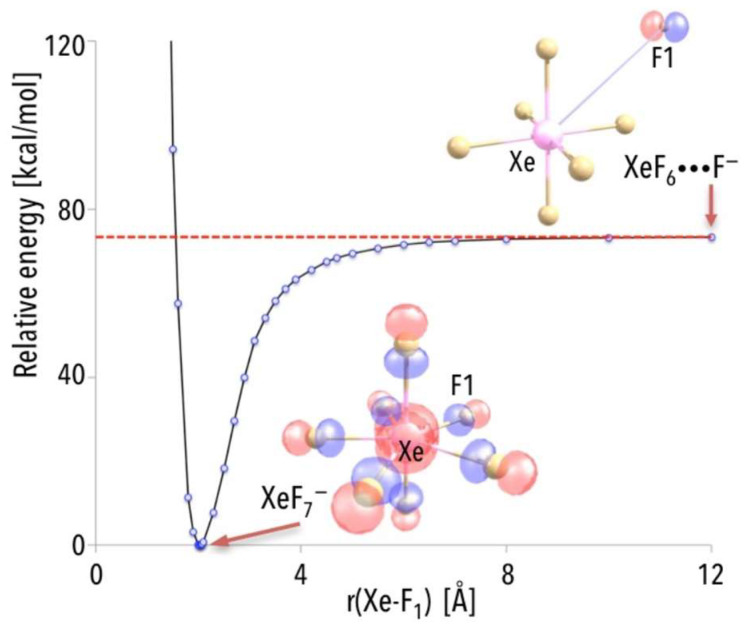
The MP2/6–311++G(3df,3pd)+ECPs energy profile for the formation of the XeF_7_^−^ anion according to the XeF_6_ + F^−^ → XeF_7_^−^ reaction. The red asymptote corresponds to the sum of the energies of isolated fragments [XeF_6_; F^−^] and reads 73.4 kcal/mol. The highest occupied molecular orbitals (HOMO) holding an additional electron are depicted for the structures corresponding to r = 2.026 Å (equilibrium geometry) and r = 12 Å. Atoms are colored yellow (F) and pink (Xe). Reproduced with permission from ref. [43]. Copyright 2016 Royal Society of Chemistry.

In [43], the mechanism of the NgF_7_^−^ anion formation by assessing the energy change that accompanies the reaction of substrates (XeF_6_ and F^−^) leading to the creation of the XeF_7_^−^ anion was explained. While analyzing the energy profile for the XeF_6_ + F^−^ → XeF_7_^−^ process, the excess electron must be assigned to F rather than to XeF_6_ (for the separated F and XeF_6_ systems, see Figure 6) due to the considerably larger electron affinity of the fluorine atom. Indeed, as indicated by the localization of the highest energy molecular orbital (HOMO), the additional electron is in the vicinity of the F species (as depicted in Figure 6). The part of the excess electron density gets transferred to the remaining F atoms as the originally distant F approaches XeF_6_ to form the XeF_7_^−^ anion (see Figure 6, where also the HOMO for the equilibrium XeF_7_^−^ structure is shown). Consequently, the additional electron is equally distributed (due to symmetry) among all F ligands in the XeF_7_^−^ anion. The energy smoothly decreases as the “extra” F atom approaches the neutral XeF_6_ molecule, and there is no barrier that must be surmounted. Since the energy of the separated XeF_6_ and F^−^ is much larger (by ca. 73 kcal/mol, see the asymptote in Figure 6) than the energy of XeF_7_^−^ anion at its equilibrium structure, and having in mind that the XeF_6_ + F^−^ → XeF_7_^−^ process is predicted to be barrier-free, one may expect the XeF_7_^−^ anion to be created spontaneously in the gas phase (whenever F^−^ ions find themselves in the vicinity of XeF_6_ molecules) [43].

**Figure 7:**
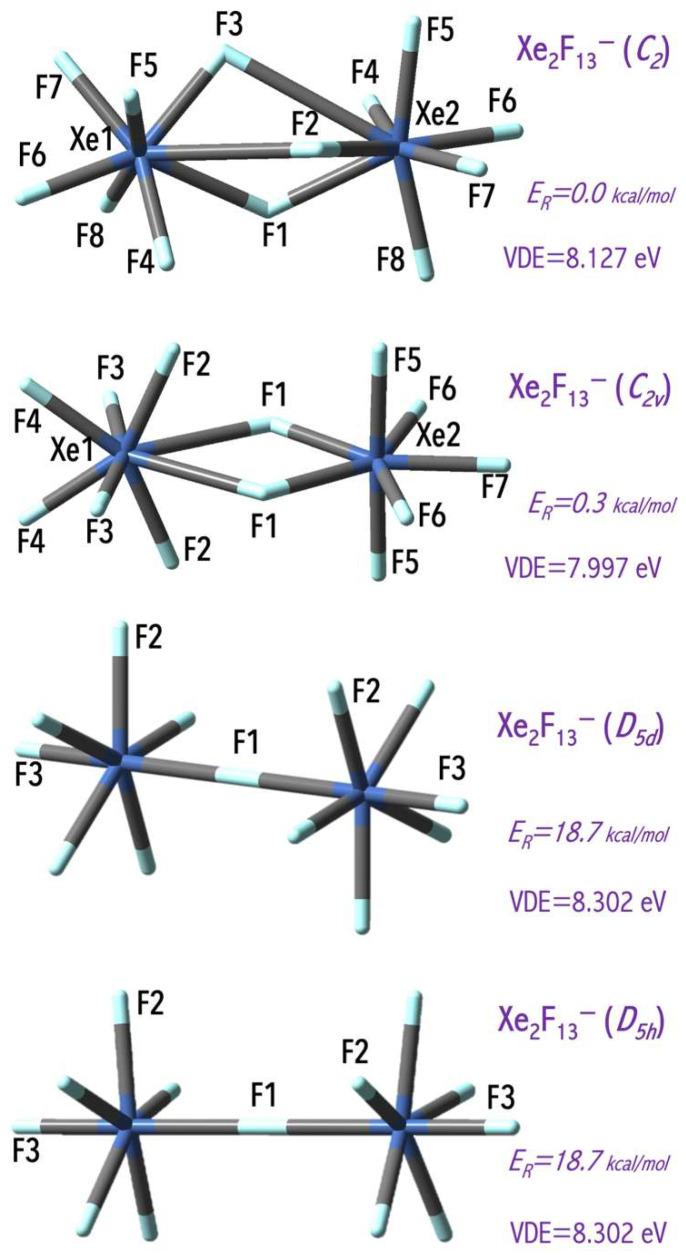
The equilibrium structures of the Xe_2_F_13_^−^ anion as obtained at the MP2/6-311++G(3df,3pd)+ECPs level. The relative energies (E_R_, in kcal/mol) are estimated with respect to the C_2_-symmetry global minimum. Adapted with permission from ref. [43]. Copyright 2016 Royal Society of Chemistry.

The values of the excess electron binding energies calculated for the Ng_n_F_6n+1_^−^ anions (Ng = Xe, Rn) span the 5.2–9.6 eV [43] range and exceed the electron affinity of the chlorine atom (3.62 eV [44]). The estimated VDE values depend on central atom use and on the number of fluorine ligands (nF, Figure 5). In general, a larger central atom (with a larger atomic mass) leads to a larger VDE value, and this observation agrees with the mathematical model for the VDE of superhalogen anion prediction [36]. In addition, the VDE of the Rn_n_F_6n+1_^−^ anions increases with the number of electronegative ligands (nF) increase due to the stabilization effect of the delocalization of excess negative charge over a larger number of ligands. Replacing mononuclear NgF_n_^−^ anions with binuclear Ng_2_F_13_^−^ systems allow for binding more ligands without system destabilization (due to the reduction of ligand-ligand repulsion in a binuclear system in comparison to a mononuclear system). In addition, my results indicate that the VDE of the binuclear superhalogen Xe_2_F_13_^−^ system depends on its geometrical structure and spans the 7.997–8.302 eV range, as shown in Figure 7 [43]. This observation conforms with the subsequent theoretical studies [45,46] indicating that VDE strongly depends on the superhalogen’s geometrical structure (if various isomeric structures are possible and stable).

### 3.3. Alternative Ligands in Superhalogen Design

**Figure 8:**
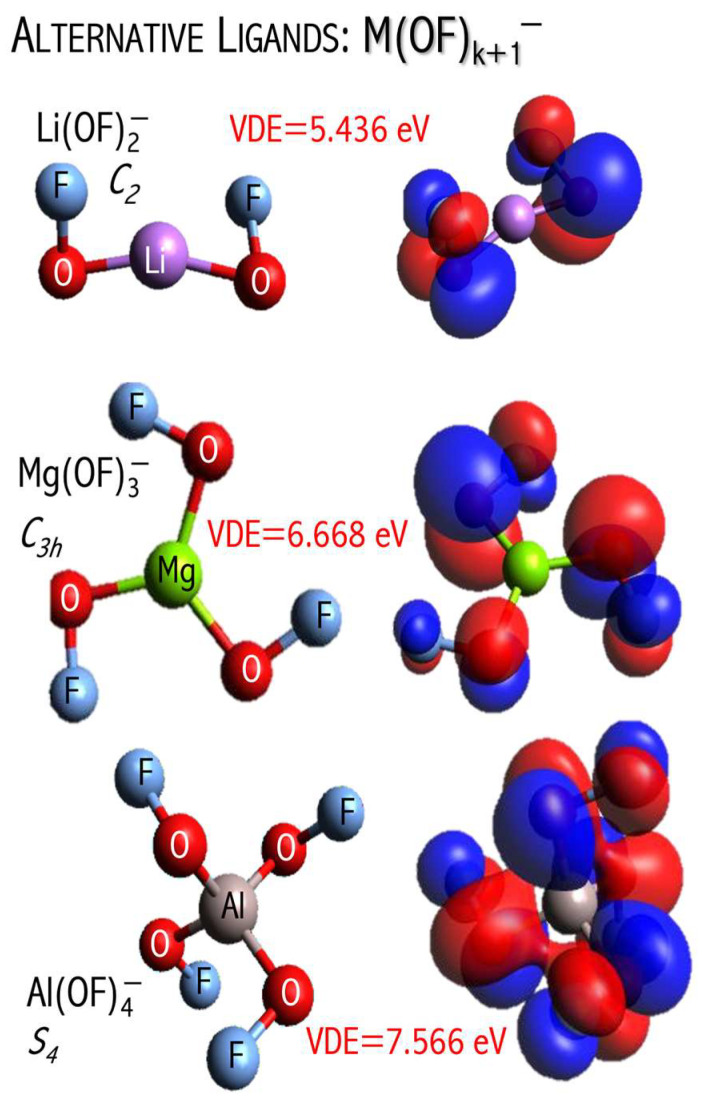
The representative ground state structures of ${M(OF)}_{k+1}^{-}$ anions as obtained at the MP2/6-311+G(d) level and corresponding highest occupied molecular orbitals (HOMOs). Adapted with permission from ref. [45]. Copyright 2015 Elsevier B.V.

**Figure 9:**
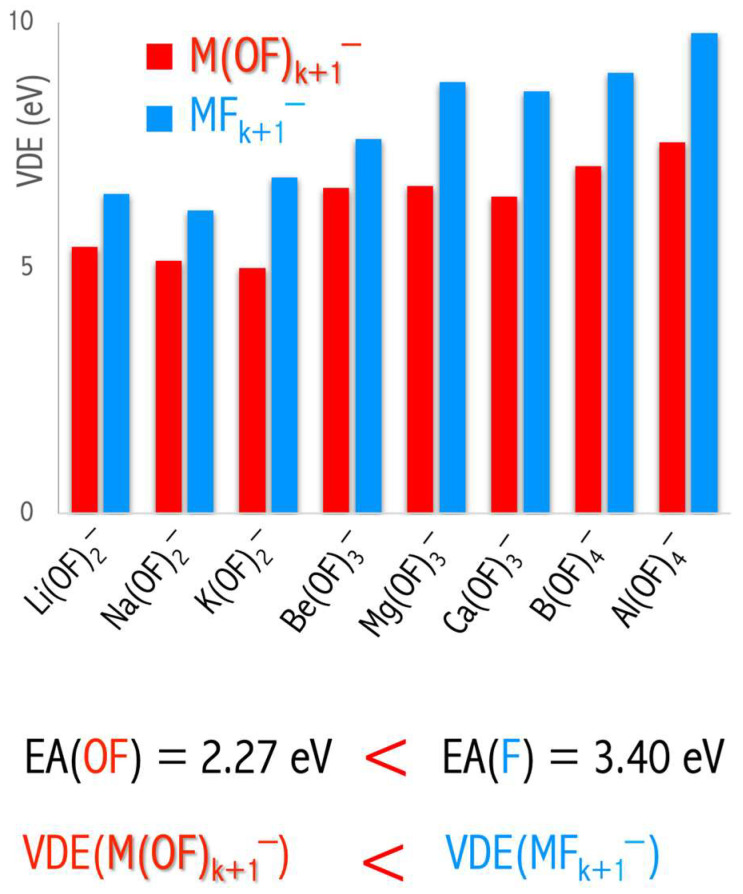
The vertical detachment energy (VDE in eV) of M(OF)_k+1_^−^ (in red) and MF_k+1_^−^ (in blue) anions as obtained from the OVGF-6-311+G(3df). The plot is based on data from ref. [45].

A typical superhalogen has its central metal atom decorated with halogen ligands [8,9,14]. However, both theoretical and experimental studies [18] have shown that the presence of metal or halogen atoms is not mandatory for a cluster to exhibit superhalogen properties. Examples include the noble-gas-based superhalogen molecules [43] and alternative superhalogen systems where fluoroxyl groups (OF) act as ligands [45]. The ${M(OF)}_{k+1}^{-}$ (M = Li, Na, K, Be, Mg, Ca, B, Al) ground states are highly symmetric structures and have large electronic stability, see Figure 8. The estimated VDEs always exceed 5 eV, which suggests the superhalogen nature of the ${M(OF)}_{k+1}^{-}$ anions. Despite large electronic stability, the ${M(OF)}_{k+1}^{-}$ anions seem to be thermodynamically unstable toward ${MF}_{k+1}^{-}$ loss/formation [45]. The thermodynamic instability of the ${M(OF)}_{k+1}^{-}$ anions might be caused by the relatively large electron binding energy of the corresponding ${MF}_{k+1}^{-}$ fragmentation products. In Figure 9, the comparison between VDE of ${M(OF)}_{k+1}^{-}$ and ${MF}_{k+1}^{-}$ anions is presented. Replacing fluorine ligands with fluoroxyl groups always leads to a VDE decrease.

The above studies indicate that the electronegativity of the ligands determines the thermodynamic stability of superhalogen anions. In Figure 9, the comparison between the VDE values of the ${M(OF)}_{k+1}^{-}\;$ and ${MF}_{k+1}^{-}$ anions is depicted. Replacing fluorine ligands with fluoroxyl groups always leads to a decrease in the VDE value. The VDE of a superhalogen anion depends mainly on the electronegativity of the ligands with which this anion is decorated. In addition, the higher VDE of an anion is observed for a large number of such ligands due to the so-called ‘collective effects’ [17]. The use of very strong electron acceptors such as fluorine ligands (EA = 3.401 eV [47]) leads to an increase in VDE when compared to the system utilizing fluoroxyl groups (EA = 2.272 eV [31]). Explicitly, in the Li(OF)_2_^−^/Be(OF)_3_^−^/B(OF)_4_^−^ and LiF_2_^−^/BeF_3_^−^/BF_4_^−^ series, the increase in VDE from 5.436 to 7.079 eV and from 6.510 to 8.975 eV [9,48], respectively (see Figure 9) [45]. Based on the ab initio computations, it was proved not only that electronegative substituents ensure large VDE but also that the difference in EA of ligands determines the thermodynamic stability of the resulting superhalogen anions [45].

### 3.4. Polynuclear Superhalogens and Mixed Ligands

The design of polynuclear superhalogen allows to enhance the electron binging energy [17,20]. Inspired by the results obtained for binuclear superhalogen anions [43], I decided to further investigate the influence of the number of central atoms (and ligands) on the electron binding energy in the resulting anions. In addition, I decided to check whether replacing fluoroxyl groups with oxygen and fluorine ligands would enhance the thermodynamic stability of the system. Based on the ab initio computations, I proved the ability to form stable superhalogen anions using mixed ligands (O, F) [46]. Moreover, replacing fluoroxyl group [45] with oxygen and fluorine ligands leads to thermodynamically stable ${{Mg}_{n}F_{2n+1-2m}O}_{m}^{-}$ (n = 2, 3; m = 1−3) anions. The positive Gibbs free energy for the ${{Mg}_{n}F_{2n+1-2m}O}_{m}^{0/-}\to {{Mg}_{n-1}F_{2n-1-2m}O}_{m}^{0/-}+{MgF}_{2}$ process indicate that each superhalogen and its anion should be formed spontaneously. It means that the $Mg(F)O^{0/-}$ or ${Mg}_{2}FO_{2}^{0/-}$ species, once synthesized, should successively attach the MgF_2_ molecules to develop polynuclear ${{Mg}_{n}F_{2n+1-2m}O}_{m}^{0/-}$ systems [46].

**Figure 10:**
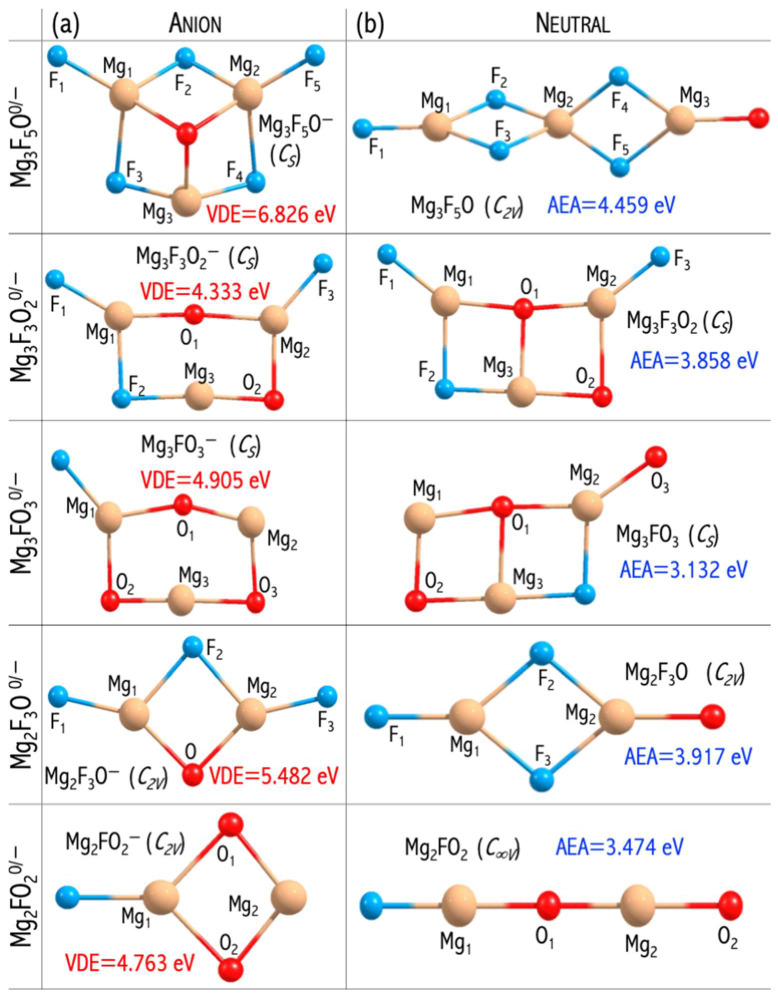
The ground state of (**a**) ${{Mg}_{n}F_{2n+1-2m}O}_{m}^{-}$ anions, and (**b**) corresponding neutral parents. The vertical detachment energy (VDE in eV) and adiabatic electron affinity (AEA in eV) were obtained from the OVGF/6-311+G(3df). Atoms are colored blue (F), beige (Mg), and red (O). Adapted with permission from ref. [46]. Copyright 2019 American Chemical Society.

**Figure 11:**
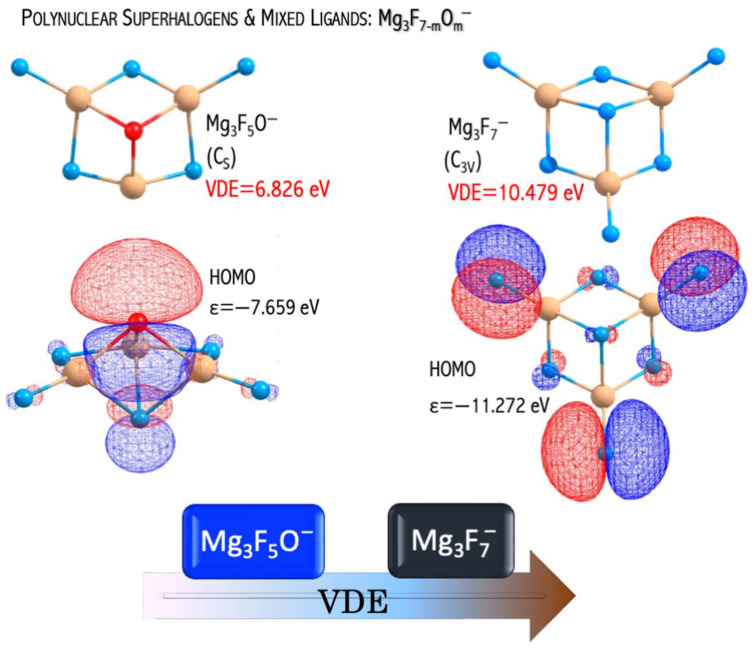
The HOMO orbital and orbital eigenvalue (ε, in eV) of the Mg_3_F_5_O^—^ (on the left-hand side) and Mg_3_F_7_^−^ (on the right-hand side) anions. Atoms are colored blue (F), beige (Mg), and red (O). Figure based on data from ref. [46,49].

The ${{Mg}_{n}F_{2n+1-2m}O}_{m}^{-}$ anions have large electronic stabilities. Their VDEs span the 4.763−6.826 eV range (Figure 10) and considerably exceed the EA of the chlorine atom (3.62 eV [44]) [46]. The obtained VDE values, albeit significant, are lower than the VDE of the non-mixed ${Mg}_{n}F_{2n+1}^{-}$ anions described in [49]. These observations can be explained by the difference in their highest occupied molecular orbital (HOMO) nature. In the case of mixed ${{Mg}_{n}F_{2n+1-2m}O}_{m}^{-}$ anions, the HOMO reveals the bonding and antibonding character with respect to the Mg−F and Mg−O interactions, respectively (Figure 11). In turn, the non-mixed ${Mg}_{3}F_{7}^{-}$ anion has a non-bonding HOMO orbital consisting purely of fluorine atomic orbitals (Figure 11). The described alteration in the HOMO nature was observed for the first time by Freza et al. [7] and explains the electronic stability decrease with mixed ligand introduction. Therefore, the replacement of fluorine atoms with mixed ligands (F, O) leads to symmetry breaking in mixed superhalogen anions and subsequent decreases in electron stability (Figure 11) [46,49].

Even though the VDEs of the designed oxyfluoride superhalogens are lower than those of the corresponding non-mixed fluoride systems (described in [49]), still, the designed superatoms do have very large VDEs (approach 7 eV) [46]. Hence, the possibility of utilizing mixed superhalogens in chemical applications should be promising and is yet to be discussed in the following Section 3.6.

The enhanced high electronic stability of the designed superatoms [43,45,46,49] is associated with the following:

(i) large electronegativity of the ligands (F, O, OF) that efficiently supports strong extra electron binding,
(ii) the large number of electronegative ligands that ensure the efficient negative charge distribution in the anion,
(iii) HOMO nature (i.e., it is mainly contributed by atomic orbitals of electronegative ligands),
(iv) the positive charges of metal cores provide extra stabilization of an electron delocalized over ligands.

The above factors have a stabilization effect on the ability of the designed systems to bind an extra electron. Overall, the Ng_n_F_6n+1_^−^ (Ng = Xe, Rn; n = 1–2) [43], ${M(OF)}_{k+1}^{-}$ ((M = Li, Na, K, Be, Mg, Ca, B, Al) [45], ${{Mg}_{n}F_{2n+1-2m}O}_{m}^{-}$ (n = 2, 3; m = 0−3) [46,49] have VDE exceeding the electron affinity of Cl atom (3.62 eV [44]) and can act as a strong oxidizing agent [49,50,51].

### 3.5. Designed Superhalogens as Strong Oxidizing Agents

The superhalogens described in the above sections have a large electron affinity. Therefore, a natural continuation of the above research was to verify the hypothesis on the ability of the designed superhalogen systems to act as oxidizing agents in chemical processes. Explicitly, the stability and the charge flow in [**D**][superhalogen] complexes, where **D** is an atom (e.g., Li) or molecule (metal oxide, C_60_ fullerene), were verified. The electron affinity of superhalogen determines its ability to ionize molecules effectively. In my studies, I considered the superhalogens whose electron binding energy (expressed by the VDE of the corresponding anion) covers the 4.8–10.5 eV range. I chose both mononuclear (LiCl_2_, LiF_2_, LiI_2_, MgCl_3_, MgF_3_, AlCl_4_, AlF_4_) [7,8,9] as well as polynuclear Mg_3_F_7_ [49] superhalogens due to their simple structure and sufficient electron affinity.

**Figure 12:**
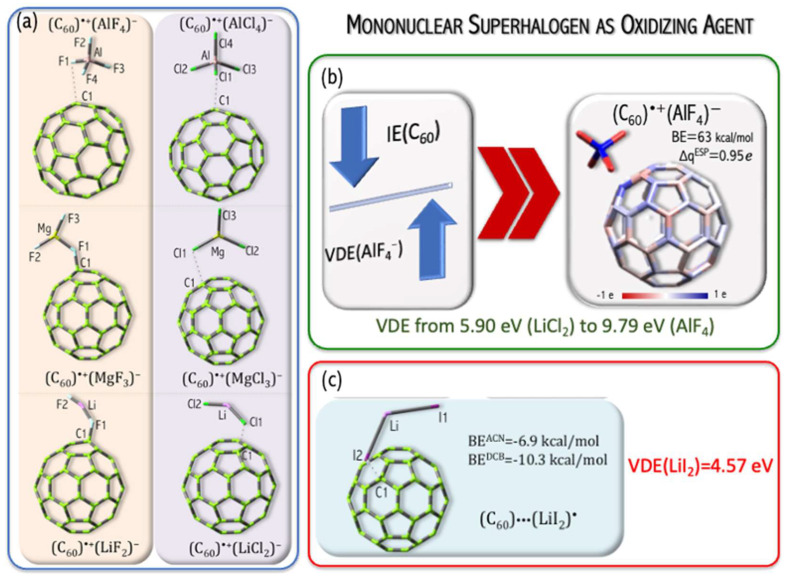
(**a**) The ground states of (C_60_)^•+^(superhalogen)^−^ ionic compounds, (**b**) the charge distribution (Δq^ESP^ in e) and binding energy (BE in kcal/mol) in the (C_60_)^•+^(superhalogen)^−^ ionic systems, (**c**) the (C_60_)···(LiI_2_)^•^ complex. Adapted with permission from ref. [51]. Copyright 2016 Royal Society of Chemistry.

I studied the influence of superhalogen type (mononuclear and polynuclear) on its ability to serve as an effective oxidizing agent for fullerene C_60_ ionization [49,51]. The C_60_ ionization (IE = 7.58 eV [52]) is, however, difficult to occur and requires a strong oxidizer. It was demonstrated that utilizing very strong oxidizing agents (such as neutral superhalogens) leads to complete or partial C_60_ nanoparticle ionization [49,51]. The fullerene, when combined with a properly chosen superhalogen (which plays the role of oxidizer), should form stable and strongly bound ionic (C_60_)^•+^(superhalogen)^−^ compounds, see Figure 12a,b. The above conclusion was formulated based on the ab initio calculations on (i) the structural deformation of superhalogens and fullerene upon the ionization process, (ii) predicted charge flow between C_60_ and each superhalogen (which allows for the estimation of the electron density flow from the nanoparticle to a superhalogen during the ionization process) and (iii) the interaction energy between the superhalogen and fullerene in the (C_60_)^•+^(superhalogen)^−^ system.

**Figure 13:**
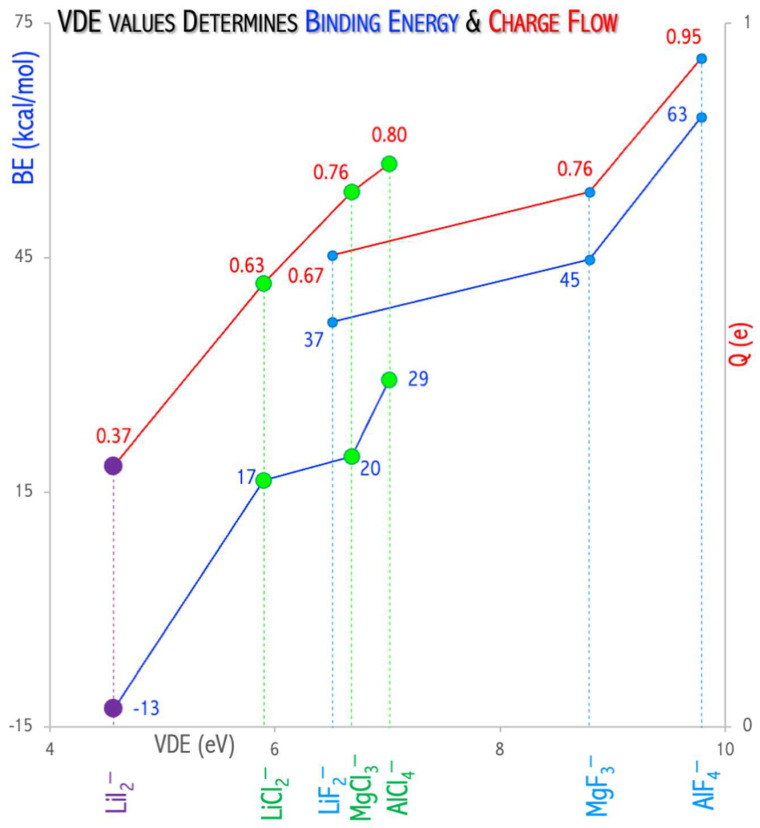
The VDE of superhalogen anion vs. binding energy (BE in kcal/mol, points connected via blue lines) and charge flow (Q in e, points connected via red lines) of C_60_/superhalogen compounds. Results obtained for compounds consisting of fluorine, chlorine, and iodine ligands are represented by blue, green, and purple circles, respectively. The plot is based on data from ref. [51].

The value of vertical electron detachment energy in the superhalogen anion determines the stability and charge flow between C_60_ and the superhalogen [49,51]. Figure 13 shows the increase in the interaction energy and charge flow with VDE increase. The process of fullerene binding by superhalogens (LiCl_2_, LiF_2_, LiI_2_, MgCl_3_, MgF_3_, AlCl_4_, AlF_4_, Mg_3_F_7_) with a VDE in the range of 5.9–10.5 eV is exothermic (the interaction energy is 19.5–62.9 kcal/mol in the gas phase, 7.9–87.4 kcal/mol for acetonitrile, 8.3–83.8 for ortho-dichlorobenzene). The (C_60_)···(LiI_2_)^•^ complex formation is an endothermic process (the interaction energy is −12.7 kcal/mol (gas phase, Figure 13), −6.9 kcal/mol (acetonitrile), −10.3 (ortho-dichlorobenzene, Figure 12c). This means that the VDE (4.8 eV) of the LiI_2_^−^ anion is insufficient to lead to the effective oxidation of the fullerene molecule, and the LiI_2_ superhalogen only forms a weakly bound complex (C_60_)···(LiI_2_)^•^, see Figure 12c [51]. On the other hand, the use of a properly selected superhalogen (having a VDE above 5.9 eV, see Figure 12a,b) leads to a strongly bonded ionic (C_60_)^•+^(superhalogen)^−^ compound [49,51].

**Figure 14:**
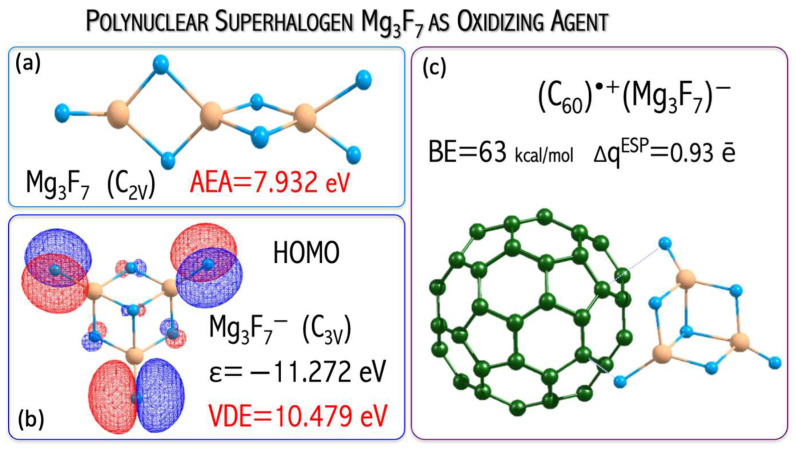
(**a**) The Mg_3_F_7_ superhalogen, (**b**) Mg_3_F_7_^−^anion, (**c**) (C_60_)^•+^(Mg_3_F_7_)^−^ ionic compound. Adapted with permission from ref. [49]. Copyright 2018 John Wiley and Sons.

**Figure 15:**
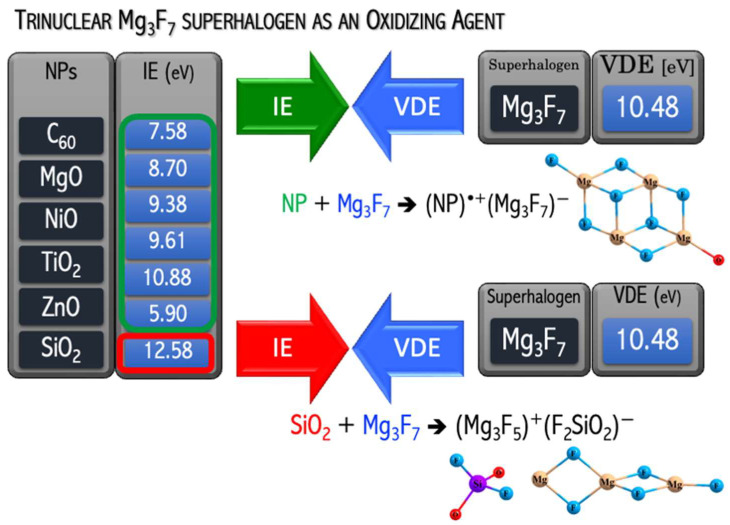
Ionization energy (IE in eV) of nanoparticle (NP) vs. vertical electron detachment energy (VDE in eV) of Mg_3_F_7_^−^ anion in NP/Mg_3_F_7_ compounds. Atoms are colored blue (F), beige (Mg), and red (O). The plot is based on data from ref. [49,50].

A natural continuation of the above research was a project in which the usefulness of the hypothesis about the possibility of binding and removing potentially toxic metal oxide nanoparticles from the environment was tested. In [49], I demonstrated that the replacement of mononuclear superhalogens by the polynuclear Mg_3_F_7_ system leads to strongly bonded (C_60_)^•+^(superhalogen)^−^ compounds, see Figure 14. In [50], I verified the ability of the polynuclear Mg_3_F_7_ superhalogen to oxidize mono- and metal dioxides (CoO, CuO, MgO, NiO, ZnO, MnO_2_, SiO_2_, TiO_2_). The verification performed for many [MeO_n_][Mg_3_F_7_] systems was positive (Figure 15). In particular, the Mg_3_F_7_ superhalogen can bind and form stable chemical compounds when interacting with metal oxides, whose ionization energy approaches 11.4 eV (Figure 15). In contrast, the Mg_3_F_7_ superhalogen is not able to effectively ionize the silica oxide SiO_2_, whose ionization energy exceeds 12 eV (Figure 15). Based on the ab initio results [50], the key role of the relationship between the ionization energy of the metal oxide and the binding energy of the excess electron of the superhalogen with which MeO_n_ interacts was indicated (Figure 15).

The ab initio studies on [**D**][superhalogen] systems [49,50,51] not only explain the stability (or lack thereof) of the [**D**][**A**] chemical compounds (**D** = C_60_, CoO, CuO, MgO, MnO_2_, NiO, SiO_2_, TiO_2_, ZnO; **A** = LiF_2_, LiCl_2_, LiI_2_, MgF_3_, MgCl_3_, AlF_4_, AlCl_4_, Mg_3_F_7_), but also they allow to predict the stability of almost any chemical systems built according to the donor-acceptor (**D**–**A**) scheme (Figure 15) and can be useful in the design of new nanomaterials (e.g., binary nano-salts, which is yet to be discussed in Section 3.10).

### 3.6. Superhalogens as Electrolytes of Lithium-Ion Batteries

**Figure 16:**
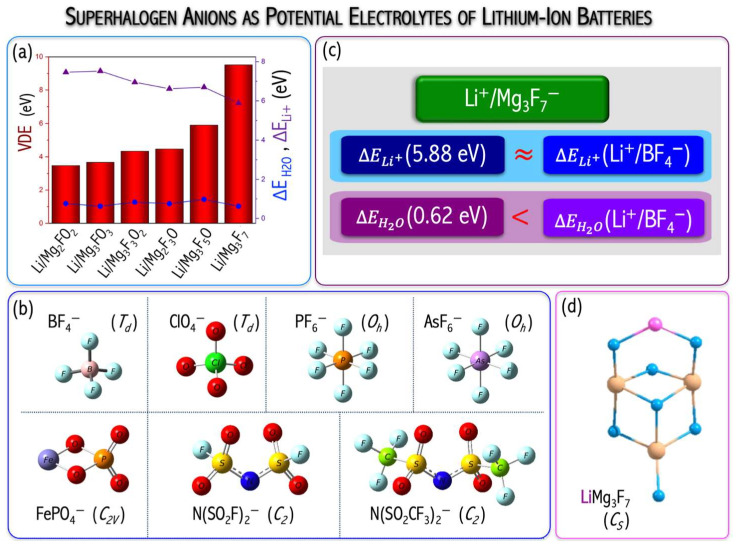
(**a**) Vertical electron detachment energy (VDE, in eV) of Mg_n_F_2n+1-2m_O_m_^−^ superhalogen anions vs. the Li-ion binding energy ($\Delta E_{{Li}^{+}}$) and sensitivity to water ($\Delta E_{H_{2}O}$) in [Li]^+^[Mg_n_F_2n+1-2m_O_m_]^−^ systems; (**b**) superhalogen electrolytes in commercial Li-ion batteries; (**c**) the Mg_3_F_7_^−^ anion as the best candidate for an electrolyte in Li-ion batteries (**d**) the ground state of [Li]^+^[Mg_3_F_7_] ^−^ salt. Adapted with permission from ref. [56]. Copyright 2019 John Wiley and Sons.

**Figure 17:**
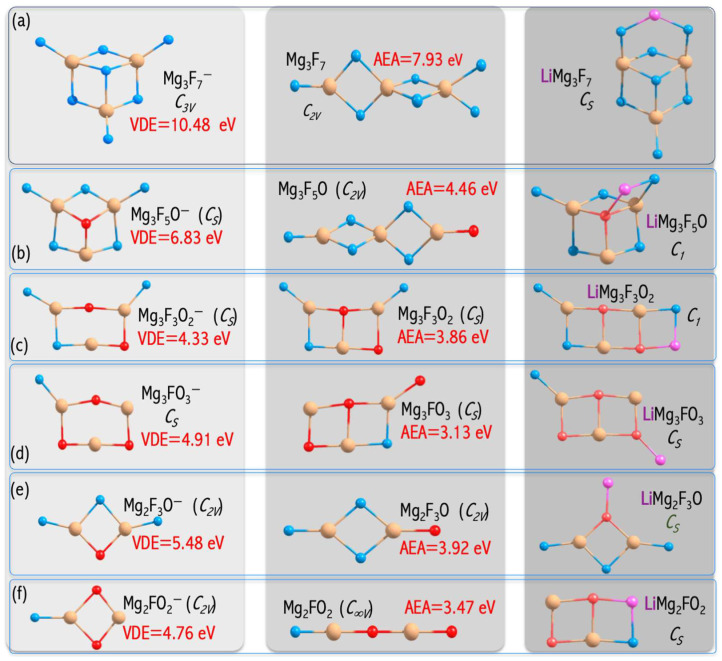
(**a**–**f**) Global minima of the Mg_n_F_2n+1−2m_O_m_^−^ superhalogen anions (**left** column), Mg_n_F_2n+1−2m_O_m_ superhalogens (**middle** column), and Li^+^/Mg_n_F_2n+1−2m_O_m_^−^ compounds (**right** column). Figure based on data from ref. [46,49,56].

Lithium-ion batteries (LIBs) have many applications—from portable electronic devices to electric vehicles. The LIBs, however, suffer from limited recycling life (<1000 cycles) as well as durability and safety issues, much of which is attributed to the current use of organic electrolytes. In this regard, the replacement of organic liquid electrolytes with inorganic electrolytes seems to be very appealing. Examples include LIBs consisting of LiBF_4_, LiAsF_6_, LiPF_6_, LiFePO_4_, LiClO_4_, or LiN(SO_2_F)_2_ inorganic electrolytes, see Figure 16b. The anionic subunits of these electrolytes are all superhalogen anions. However, superhalogen electrolytes have some limitations. LiBF_4_ has the inferior ability to form solid electrolyte interphases (SEIs), LiClO_4_ is explosive, while LiAsF_6_ is poisonous [53]. LiPF_6_ decomposes to PF_5_ and LiF, whereas PF_5_ is readily hydrolyzing to form PF_3_O and HF. PF_3_O and HF are very reactive on the cathode and anode surfaces, which negatively impacts the electrode performance [54]. LiFePO_4_ suffers from the so-called ‘memory effect’ resulting in losing the usable capacity if recharged repeatedly after being only partially discharged [55]. Besides, LIBs have limited performance at elevated temperatures and limited life cycles (due to surface phenomena on both electrodes). In this frame, the performance of the polynuclear magnesium-based superhalogen anions (1st column of Figure 17) [46,49] as potential alternatives for electrolytes of lithium-ion batteries was tested.

For this purpose, I designed and examined the stability of the Li^+^/Mg_n_F_2n+1−2m_O_m_^−^ (n = 2, 3; m = 0–3) compounds and corresponding ground states are provided in Figure 17. Based on the ab initio calculations performed both in the gas phase and water solution, I demonstrated that polynuclear superhalogen anions can act as novel electrolytes in lithium-ion batteries. This conclusion is supported by the following:

(i) The geometrical stability of Li^+^/Mg_n_F_2n+1−2m_O_m_^−^ equilibrium structures (Figure 17).
(ii) Hardly any charge transfer (below 0.01 e) between lithium ion and each of superhalogen Mg_n_F_2n+1−2m_O_m_^−^ anions confirming the superhalogen anion stability in lithium salt due to negligible electronic density transfer from Li^+^ upon its interaction with superhalogen anion.
(iii) Vertical ionization potential (VIP) increasing upon Li^+^ counterion introduction. The Li^+^ counterion introduction results in more stable Li^+^/Mg_n_F_2n+1−2m_O_m_^−^ compounds (VIP^CCSD(T)^ = 7.8–12.2 eV [56]) in comparison to corresponding anions (VDE^CCSD(T)^ = 3.471–9.515 eV [46,49]).
(iv) The interaction energy between the Li^+^ cation and a superhalogen anion ($\Delta E_{{Li}^{+}}$) covering the 5.884–7.456 eV range (in a vacuum, Figure 16a) and 0.365–0.750 eV range (in water solution) [56]. The lower $\Delta E_{{Li}^{+}}$ lithium ion bonding the higher Li^+^ ion conductivity.
(v) The low binding energy of H_2_O to the electrolyte ($\Delta E_{H_{2}O}$ = 0.616–0.969 eV, Figure 16a) as a criterium of chemical stability of electrolyte toward interaction with water molecules.
(vi) The large electrolyte stability window (EW of 9.03–14.70 V [56]) ensures maximal potential difference between electrodes. The high operating voltage of the cell is achieved if the LUMO and HOMO levels of the electrolyte are separated by a large band gap.

Based on the ab initio computations and detailed analysis, a general recommendation on polynuclear superhalogen anion performance as an electrolyte in lithium-ion batteries was formulated. From the ab initio calculation result (performed both in a vacuum and with the PCM solvation model) analysis, the magnesium-based superhalogens Mg_n_F_2n+1−2m_O_m_ (n = 2, 3; m = 0–3) with high electron binding energies (manifested by the vertical electron detachment energies (VDEs) of their corresponding daughter anions of the 4.76–10.48 eV range, see Figure 17) might serve as building blocks of lithium salts. Since the Li-ion binding energy of the Li^+^/Mg_3_F_7_^−^ salt ($\Delta E_{{Li}^{+}}$ = 5.884 eV, Figure 16c) and sensitivity to water ($\Delta E_{H_{2}O}\;$= 0.618 eV, Figure 16c) were found to be lower compared to those of several currently used electrolytes in commercial Li-ion batteries (e.g., LiFePO_4_), the Mg_3_F_7_-based electrolyte is recommended for lithium-ion battery application [56]. The analysis of potential ion battery electrolytes from the superhalogen perspective [56] opens a path for the design and synthesis of desirable superatomic systems as promising building blocks for new functional materials.

### 3.7. Superatoms as Building Blocks of Materials

Designing novel superatoms is aimed at proposing the building blocks for functional materials. The main benefit of replacing conventional bulk species with superatom-based materials is the possibility of tuning the properties of superatom. The resulting superatomic materials with unique and tailored properties extend the scope of materials science with desirable features (following the safe-by-design idea). Recently, Roy et al. [57] synthesized the binary assembly of metal chalcogenide clusters (i.e., Co_6_Se_8_(P(C_2_H_5_)_3_)_6_ (**1**A), Cr_6_Te_8_(P(C_2_H_5_)_3_)_6_ (**2**A), and Ni_9_Te_6_(P(C_2_H_5_)_3_)_8_ (**3**A)) with C_60_ carbon clusters as building blocks. The [Co_6_Se_8_(P(C_2_H_5_)_3_)_6_][C_60_]_2_ and [Cr_6_Te_8_(P(C_2_H_5_)_3_)_6_][C_60_]_2_ solids resemble conventional [M^2+^][X^−^]_2_ solids of the CdI_2_ structure type. In turn, the Ni_9_Te_6_(P(C_2_H_5_)_3_)_8_, as a more metal-rich system, possesses a larger reducing ability than Co_6_Se_8_(P(C_2_H_5_)_3_)_6_ and Cr_6_Te_8_(P(C_2_H_5_)_3_)_6_ clusters and forms cubic crystals analogous to M^+^X^−^ salts (like the natural rock salt does). Different electronic properties accompany crystallographic dissimilarity among these solids, as the **1**A•2C_60_ and **2**B•2C_60_ are gapped semiconductors, whereas **3**A•C_60_ has ferromagnetic characteristics. Even though the experimental results confirmed the ability of molecular clusters to act as building blocks of solid-state materials, they did not explain the atom-like phenomena of molecular clusters.

**Figure 18:**
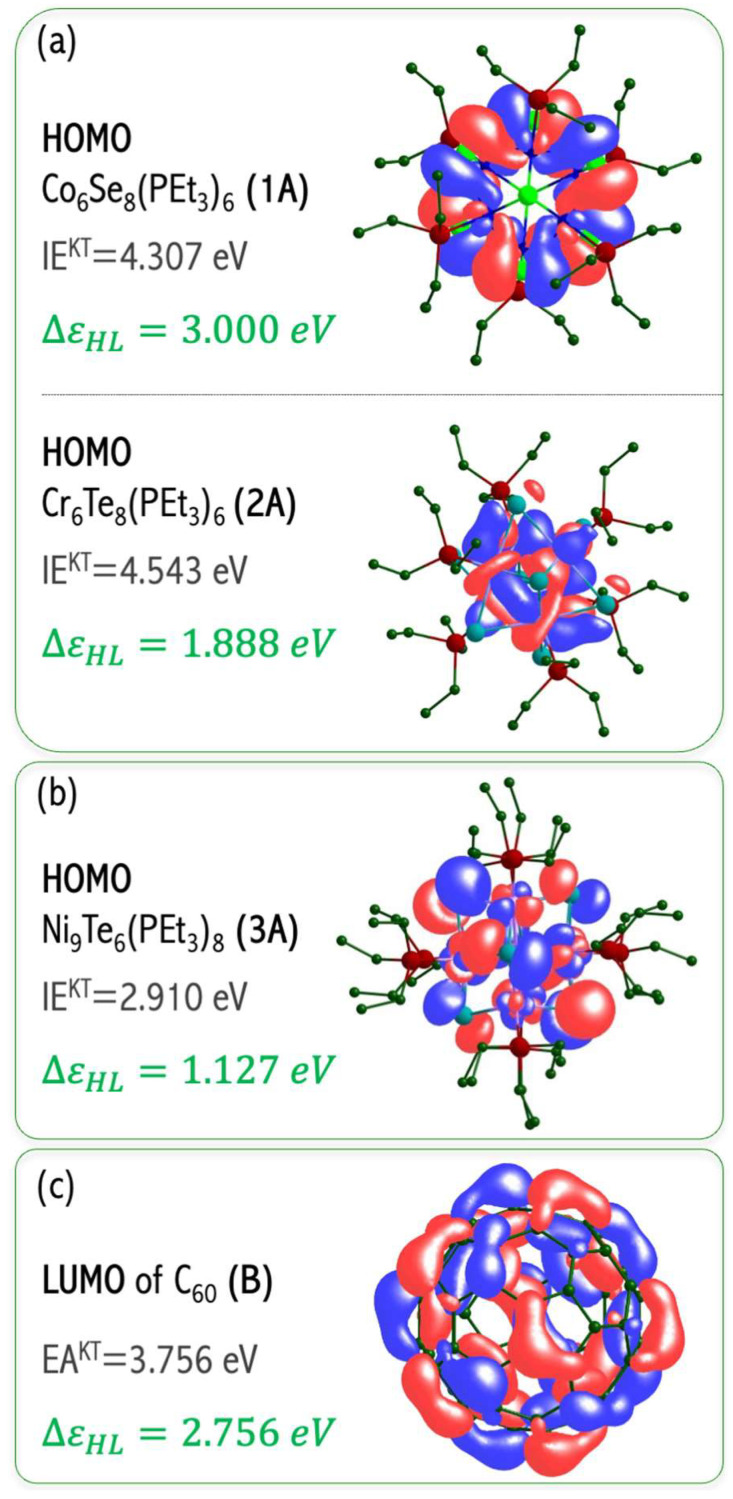
(**a**) HOMO orbital of the **1**-**2**A systems, (**b**) HOMO orbital of the **3**A superalkali, (**c**) LUMO orbital of C_60_. Adapted with permission from ref. [58]. Copyright 2020 John Wiley and Sons.

**Figure 19:**
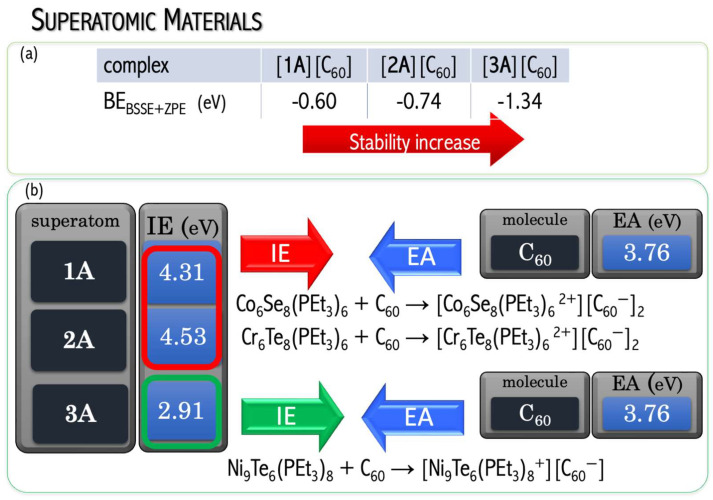
(**a**) The B3LYP-D3/DGDZVP+ECP binding energy of **1**–**3**AB systems corrected for BSSE (basis set superposition error) and ZPE (zero-point energy); (**b**) the influence of ionization energy (IE) of the metal cluster on the crystal structure of superatomic material. The plot is based on data from ref. [58].

The obtained DFT results proved the effectiveness of first-principles calculations to access the atom-like nature of transition metal clusters and to evaluate the influence of the superatomic nature on the crystal structure and physicochemical properties of cluster-assembled materials. In particular, the superalkali nature of the **3**A cluster was proved. A superalkali is a cluster of atoms exhibiting enormously low ionization energy (IE lower than the IEs of alkali metal atoms). The superalkali nature of the **3**A superatom (Ni_9_Te_6_(P(C_2_H_5_)_3_)_8_) has been proved by low IE (of 2.910 eV that is lower than the IE of cesium atom (IE = 3.9 eV [34]) and antibonding HOMO orbital in relevance to Ni–Te interactions, see Figure 18b [58]. The antibonding character of the HOMO orbital makes the molecule more susceptible to the ionization process (by lowering its IE value). In turn, the remaining metal clusters have larger electronic stabilities due to IE(**1A**) = 4.307 eV and IE(**2A**) = 4.543 eV and the bonding HOMO orbital with respect to Co–Se (**1A**) i Cr–Te (**2A**) interactions, see Figure 18a [58]. In the **1**A and **2**A clusters, the outermost electrons are more tightly bound, making these superatoms less electron-donating than chemically reactive **3**A superalkali. Consequently, **1**A and **2**A form [M^2+^][X^−^]_2_ salts of CdI_2_-type crystallographic structure, while strong electron donor **3**A agent transfers an electron to C_60_ fullerene to form [Ni_9_Te_6_(P(C_2_H_5_)_3_)_8_]^+^[C_60_] ^−^ salt (in analogy to the natural rock salt, see Figure 19).

In [58], we proved the effectiveness of the first-principles approach to assessing the activation energy of cluster-assembled materials. The energy gap between the HOMO and LUMO orbitals ($\Delta \varepsilon_{HL}$ = ε_LUMO_ − ε_HOMO_) is a measure of kinetic stability. A large $\Delta \varepsilon_{HL}$ implies high kinetic stability and low chemical reactivity due to the energetic disadvantage of adding electrons to a high-lying LUMO or removing electrons from a low-lying HOMO and so to create the activated complex of any potential reaction. The $\Delta \varepsilon_{HL}$ values of isolated components (1.13–3.00 eV, see Figure 18a–c) explain both the relatively high reactivity of the **3**A superatom ($\Delta \varepsilon_{HL}$ = 1.13 eV) and low BE of C_60_/C_60_ dimer (BE = 0.31 eV, $\Delta \varepsilon_{HL}$ = 3.00 eV). The $\Delta \varepsilon_{HL}$ is closely correlated to band gaps in solid states. The **1**A•2C_60_ and **2**A•2C_60_ systems have been proven to possess good electrical conductance with a semiconducting behavior, their Arrhenius activation energies (E_a_) being of about 150 meV (**1**A•2C_60_) and 100 meV (**2**A•2C_60_) [57]. Considering the obtained $\Delta \varepsilon_{HL}$ (68–89 meV [58]), **1**–**2**AB systems are small-gap semiconductors. The applied B3LYP-D3 functional is expected to underestimate the $\Delta \varepsilon_{HL}$, which then correlates nicely with the experimental activation energies.

**Figure 20:**
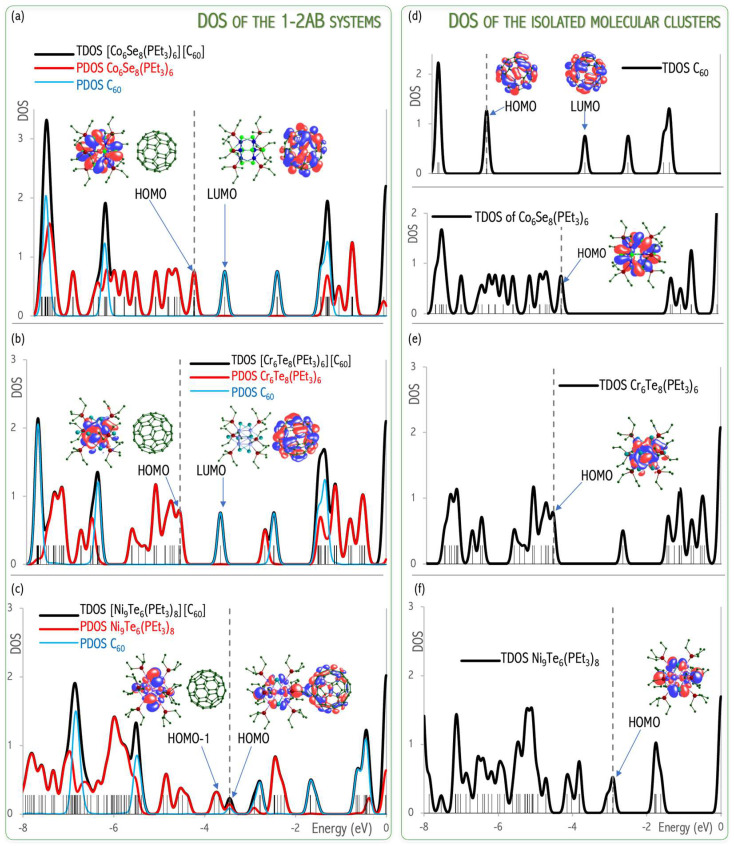
Total (TDOS) and partial (PDOS) density of states (**a**–**c**) of the **1**–**3**AB systems and (**d**–**f**) isolated clusters obtained at the B3LYP-D3/DGDZVP+ECPs level. Adapted with permission from ref. [58]. Copyright 2020 John Wiley and Sons.

The **1**–**3**AB systems show a monotonic decrease in $\Delta \varepsilon_{HL}$ values with a decrease in the ionization energy of metal clusters. All systems (**1**–**3**AB) show a reduction in $\Delta \varepsilon_{HL}$ values as compared to pure **1**–**3**A species. This $\Delta \varepsilon_{HL}$ reduction is the largest for the **3**AB system (to 0.48 eV) and gives evidence for the generation of a new HOMO orbital between the original HOMO and LUMO orbitals; consequently, the original HOMO of **3**A cluster becomes HOMO-1 and the $\Delta \varepsilon_{HL}$ becomes significantly smaller than in the pure **3**A metal cluster [58]. This means that the **3**A superalkali shifts the valence electron to C_60,_ and a new energy level is generated into the occupied orbital site. A superalkali, as a strong electron donor, enables the $\Delta \varepsilon_{HL}$ reduction of their complexes, which enhances their conductance properties [59]. In addition, Figure 20a,b of DOS shows that the states of the **1**–**2**AB systems exhibit a very localized nature. Their HOMOs are contributed atomic orbitals of the metal cluster and thus not by the anion (C_60_), as would be expected by an ionic solid. This observation implies a minor ionic character of the **1**–**2**AB chemical systems. On the contrary, the **3**AB reveals that the HOMO consists of hybridized **3**A superalkali and C_60_ orbitals forming covalent-like **3**A-C_60_ interaction, see Figure 20c [58,60]. These results proved the effectiveness of first-principles calculations to assess the influence of superatomic nature on the crystallographic structure and physicochemical properties of cluster-assembled materials.

### 3.8. Superalkali Substitution in Perovskite

The ABX_3_-type perovskites (where A and B are cations, while X is an anion) receive attention as efficacious light absorbers for solar cells due to their high-power conversion efficiency (up to 24%). The photoelectric conversion efficiency is determined by a suitable band structure. Cation (A) substitution can be an efficient approach to adjust the electronic band structure of lead halide CsPbBr_3_ perovskites. The examples include introducing superalkali cations to replace the Cs^+^ cation in the CsPbBr_3_ material [61]. The bimetallic superalkalis (LiMg, NaMg, LiCa, and NaCa) were chosen since they are structurally simple compounds and have a strong tendency to detach one electron (IE = 4.57–4.92 eV [62]) to achieve a closed-shell cation. Based on the first-principles calculations, it was evaluated whether the A-site cation may fit within the cavities in the BX_3_ framework based on the Goldschmidt tolerance factor (*α*, Equation (5)):

(5) $$\alpha =\frac{r_{A}+r_{X}}{\sqrt{2}\left(r_{B}+r_{X}\right)}$$

where

$r_{A}$ is the ionic radius of the A-cation,

$r_{B}$ is the ionic radius of the B-cation,

$r_{X}$ is the ionic radius of the anion.

**Figure 21:**
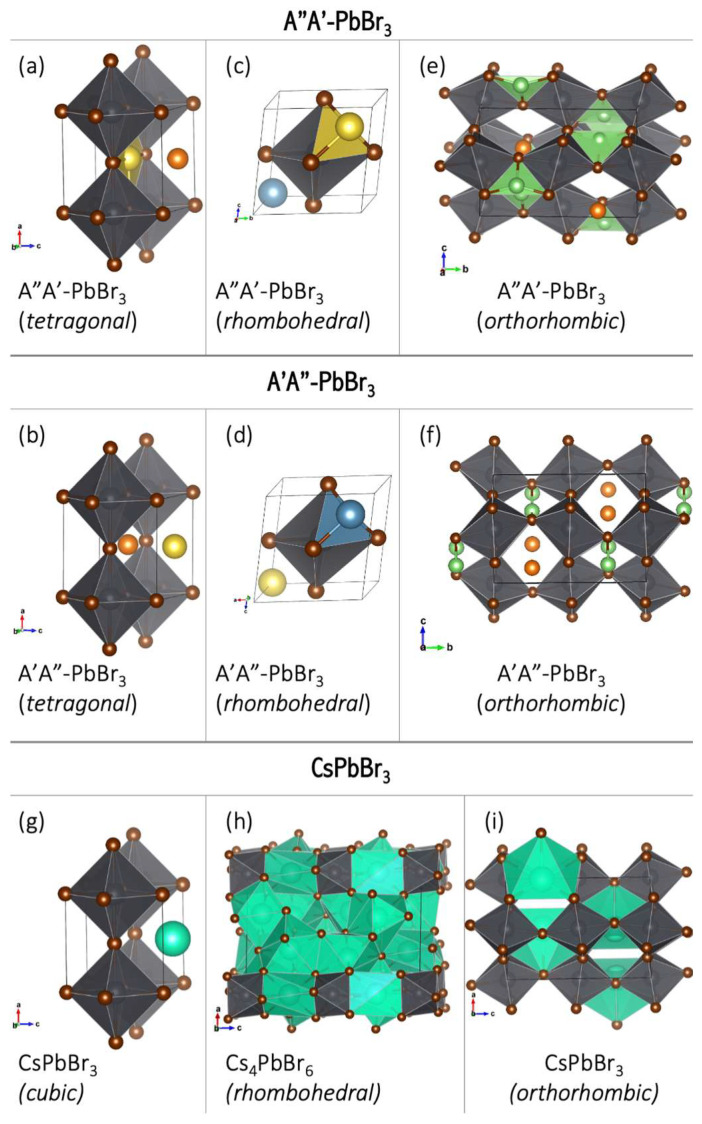
Representative structures of (**a**,**c**,**e**) A”A’–PbBr_3_ and (**b**,**d**,**f**) A’A”–PbBr_3_. The (**g**) cubic and (**i**) orthorhombic phases of CsPbBr_3_ and (**h**) Cs_4_PbBr_6_ rhombohedral phases are also provided for comparison. Atoms are colored green (Li), yellow (Na), aquamarine (Cs), orange (Mg), blue (Ca), gray (Pb), and brown (Br). A’ and A” represents alkali metal and alkaline earth metal atoms, respectively. Adapted with permission from ref. [61]. Copyright 2021 AIP Publishing.

The perovskite structure is favored by the following tolerance factors: *α* = 0.90–1.0 for cubic perovskites and *α* > 1.00 for tetragonal structures [63]. The obtained tolerance factors are 0.94 (MgLi–PbBr_3_), 1.03 (NaMg–PbBr_3_), 1.04 (CaLi–PbBr_3_), and 1.07 (CaNa–PbBr_3_), respectively, implying that the bimetallic superalkalis are a viable substitute for cesium in stable perovskites. The perovskite structure is, however, distorted from a cubic structure to a tetragonal, rhombohedral, or orthorhombic structure (Figure 21). This distortion arises because of the modified size of the A ion, which causes a tilting of the BX_6_ octahedra to optimize A–X bonding [33]. Because all superalkali cations considered in [61] are significantly less electropositive (the electron binding energy in the 4.57–4.92 eV range [14]) than cesium (IE = 3.9 eV) [34], the E_g_ values of superalkali–PBBr_3_ are much lower than that of CsPbBr_3_ (E_g_ of 2.48 eV) [28]. Based on DFT calculations, LiMg^+^ and NaMg^+^ superalkalis with smaller ionic radii (2.21 and 2.61 Å, respectively) form semiconductors with a band gap from 0.25 to 1.54 eV. The superalkalis with larger ionic radii (i.e., LiCa^+^ and NaCa^+^) create systems with a metallic nature. Hence, bimetallic superalkalis with ionic radii up to 2.6 Å can form stable perovskites with low band gaps, whereas larger superalkalis (i.e., ionic radii approaching 2.7 Å) form metallic systems.

**Figure 22:**
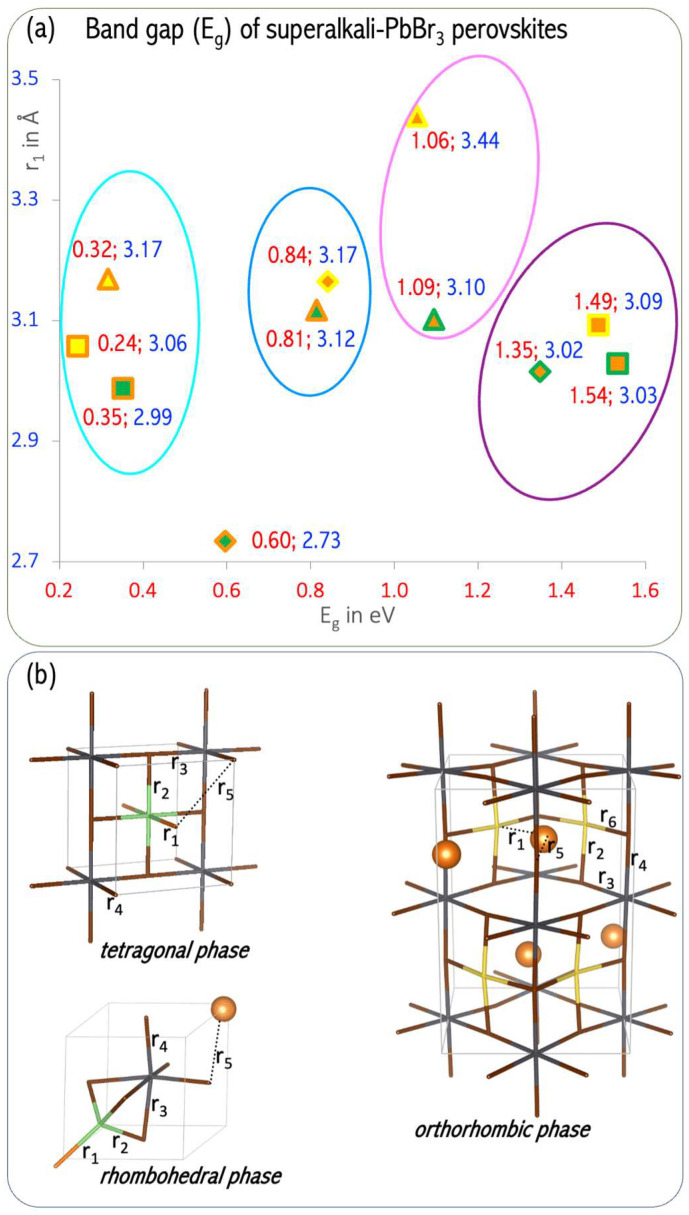
(**a**) HSE06 band gap (E_g_, in eV) values vs. A′–A′′ distances (r_1_, in Å) in superalkali–PBBr_3_ perovkites. Squares, diamonds, and triangles represent tetragonal, rhombohedral, and orthorhombic phases, respectively. The shape fill represents the atom located in the center of the lattice, while the shape outline corresponds to the counterpart atom of A′A′′ superalkali. A-site atoms are colored green (Li), yellow (Na), and orange (Mg). (**b**) Schematic structures of superalkali-PbBr_3_ phases with the bonds numbering. Adapted with permission from ref. [61]. Copyright 2021 AIP Publishing.

Based on a detailed analysis of the relationship between the length of the alkali metal–alkaline-earth metal (A′–A″) bond and the band gap (E_g_), we formulated recommendations for the modification of the electronic structure and the design of perovskites with desirable properties [61]. The lowest E_g_ value (0.24–0.35 eV) can be obtained for A′Mg–PbBr_3_ perovskites with A′–A″ bond lengths in the range of 2.99–3.70 Å (marked in cyan in Figure 22). In turn, the highest value of E_g_ (1.35–1.54 eV, marked in violet in Figure 22) can be expected for MgA′–PbBr_3_ systems with a very narrow A′–A″ bond range (3.02–3.09 Å [61]). The band gap width of 0.8 eV corresponds to the narrow A′–A″ bond range of 3.12–3.17 Å (circled in blue in Figure 22) corresponding to the orthorhombic structure of LiMg–PbBr_3_ and the rhombohedral structure of MgNa–PbBr_3_. The orthorhombic MgA′–PbBr_3_ structures correspond to an E_g_ in the range of 1.06–1.09 eV (circled in magenta in Figure 22), for which the A′–A″ distances differ by 0.34 Å. The analysis of the structure–band gap width relationship demonstrated the usefulness of superalkalis for designing perovskites with the desired photoelectric properties.

**Figure 23:**
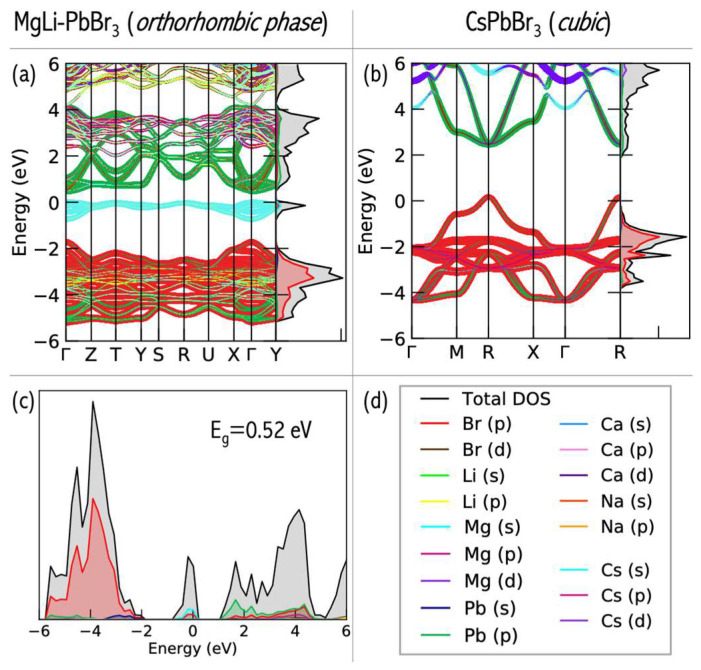
Electronic band structure and DOS for (**a**) orthorhombic phase of MgLi–PbBr_3_ and (**b**) cubic phase of CsPbBr_6_; (**c**) Density of states (DOS) for orthorhombic phase of MgLi–PbBr_3_ as obtained from HSE06; (**d**) atomic orbitals representation. Adapted with permission from ref. [61]. Copyright 2021 AIP Publishing.

A significant consequence of superalkali insertion is band structure modification in the resulting perovskites [61]. The CsPbBr_3_ perovskite has the cesium states very deep inside the conduction band (more than 2 eV above the band edge, see Figure 23b) and is not expected to play any significant role in the light adsorptions and to succeed in electronic processes. The introduction of the diatomic superalkali replacing the Cs atom in CsPbBr_3_ leads to the formation of a new band in the energy range of −2 to 0 eV in all A′A″–PbBr_3_ species (see Figure 23a), as compared to CsPbBr_3_. Within 1 eV below the valence band edge, these are ascribed to the alkali earth metal states. The A-site atom offers the main contributions for the valence band maximum (VBM) states of superalkali–PbBr_3_ compounds, which can prevent the degradation of the PbBr_3_ basic frame. Consequently, the transfer of electrons occurs at the A-site cation rather than halogen site atoms, which implies that the latter is not activated under light illumination [32]. Since halogen site atoms are not involved in the VBM states and are not activated under UV light illumination, the superalkali–PbBr_3_ compounds can be stable under these conditions.

The DFT results allowed us to formulate recommendations regarding the tunning of electronic properties of CsPbBr_3_ perovskites upon superalkali substitution [61]. The obtained band gap values strongly depend on the superalkali used and the structure formed. The electronic states of the A-site cations can be extended and directly contribute to the frontier orbitals of the lead bromide perovskite via A-site superalkali substitution, which provides electronic tunability to the band edge electronic configuration. Inserting diatomic superatomic species at the cationic A-sites of CsPbBr_3_ reduces the band gap of lead halide perovskites. More specifically, the use of LiMg and NaMg superalkalis leads to band gaps in the 0.24–1.54 eV range. The band gaps of MgLi–PbBr_3_ (1.35 eV) and MgNa–PbBr_3_ (1.06 eV) are lower than the E_g_ of CsPbBr_3_ (2.48 eV) and within the optimal E_g_ for single-junction solar cells. The E_g_ of 1.35 eV makes MgLi–PbBr_3_ a promising candidate as a light absorber for solar cell applications. The above results [61] constitute the first step for the development of novel perovskite-like materials with desirable electronic properties.

### 3.9. Novel Polynuclear Superalaklis

**Figure 24:**
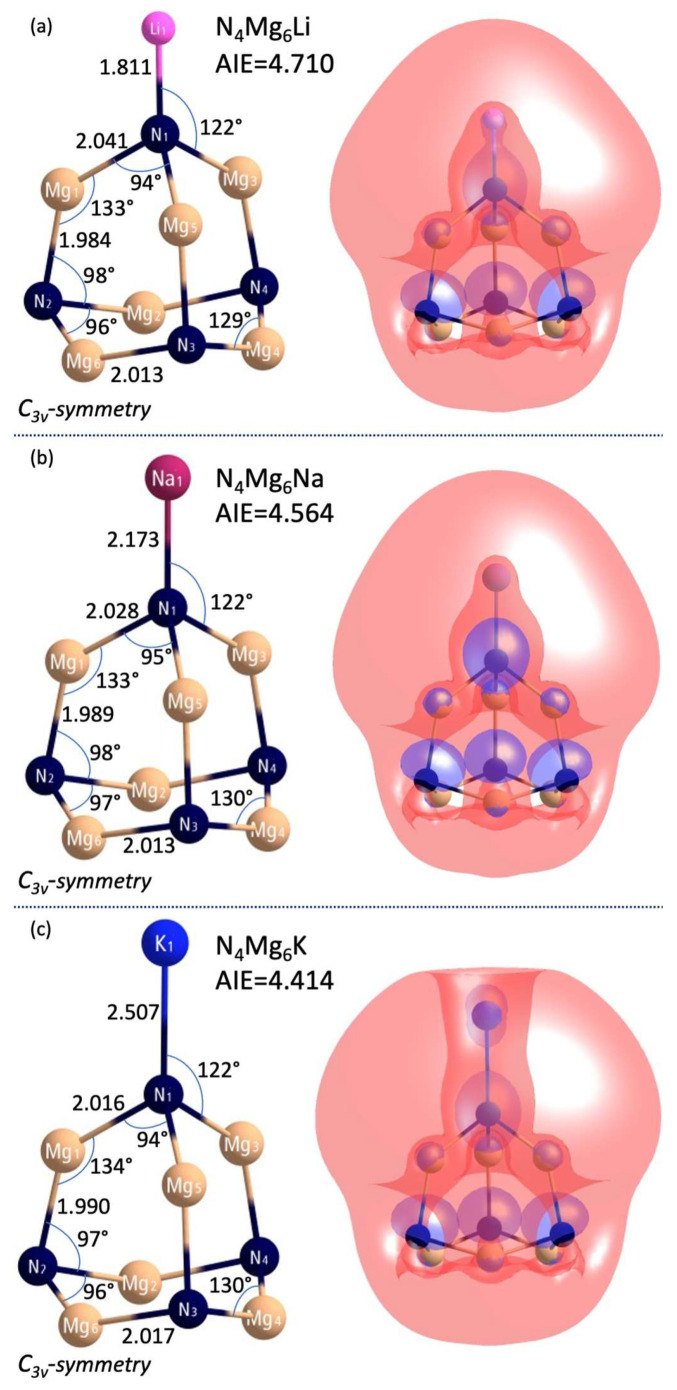
The MP2/6-311+G(d) ground states of (**a**) N_4_Mg_6_Li, (**b**) N_4_Mg_6_Na, and (**c**) N_4_Mg_6_K superalkalis and their HOMOs (plotted with a fraction of electron density equal to 0.8). Adiabatic ionization energy (AIE) in eV and bond length in Å. Adapted with permission from ref. [69]. Copyright 2020 AIP Publishing.

In 1982, Gutsev and Boldyrev introduced the simple formula **ML_k+1_** to describe one class of superalkalis, where **M** is a k-valent electronegative central atom ligated with k + 1 alkali-metal atoms (**L**) [25]. The first superalkali system described in the literature superalkali system was Li_3_O, whose experimental ionization energy (IE = 3.54 ± 0.3 eV [64]) is lower than the IE of the cesium atom (3.89 eV). Its geometrical structure and ionization energy were investigated by quantum-mechanics calculations (adiabatic and vertical ionization energies were estimated to be in the 3.45–3.60 eV range [65,66]). Typical examples of superalkalis are FLi_2_ [67] OLi_3_, ONa_3_, OK_3_ [5], NLi_4_ [66], and CLi_5_ [6]. More recently, a lot of effort has been devoted to proposing alternative superalkali species, including lithium-based polynuclear superalkalis [68], aromatic superalkali species [32], and organo-zintl clusters (such as P_7_R_4_) [33]. Despite these achievements, it is still desirable to obtain novel superalkali species to seek even lower ionization energy. The M_n_L_n·k+1_ polynuclear superalkali design seems to be the most promising direction to obtain efficient reducers. The examples include the N_4_Mg_6_M (M = Li, Na, K) polynuclear superalkalis, where four electronegative nitrogen central atoms are decorated with six electropositive magnesium atoms and one alkali metal atom (see Figure 24).

The adiabatic ionization energies obtained at the CCSD(T)/6-311+G(3df) level of theory read 4.710 (N_4_Mg_6_Li), 4.564 (N_4_Mg_6_Na), and 4.414 eV (N_4_Mg_6_K) and are smaller than those of the sodium atom (5.14 eV at the same theory level), which emphasize their superalkali nature. This conclusion is also confirmed by their large HOMO–LUMO gaps (3.662–3.958 eV), binding energies per atom (3.05–3.12 eV), and the strong ionic interactions between electronegative nitrogen atoms and electropositive metal atoms. The HOMOs of N_4_Mg_6_M superalkalis are highly delocalized over the whole clusters to reduce the repulsion among the electrons, see Figure 24. The highly diffuse HOMO also implies that the extra electron is loosely bound by the nuclei, giving rise to the low ionization energy. Also, the high thermodynamic stability of the designed superalkalis is due to the strong bonding character between metal atoms and the antibonding nature of the nitrogen-metal atoms interaction (lowering the ionization energy). Consequently, the N_4_Mg_6_M clusters have excellent reducing potential and can be used in the synthesis of charge-transfer salts and to activate moderately reactive molecules.

**Figure 25:**
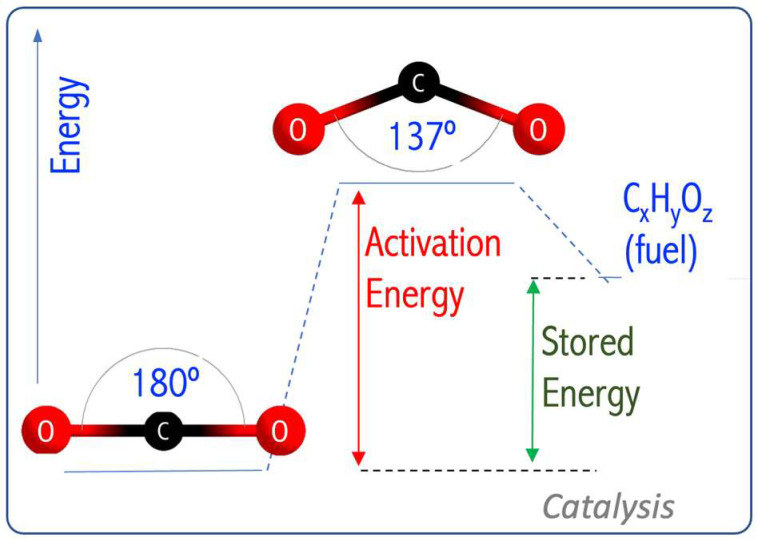
CO_2_ activation and its transformation into fuel.

Carbon dioxide (CO_2_), a greenhouse gas, is a cheap, nontoxic, and abundant carbon feedstock. So, developing efficient catalysts that convert CO_2_ into fuel and value-added chemicals is vital to reduce the growing energy crisis and global warming. CO_2_ can also be transformed into one-carbon (C_1_) molecules (e.g., formic acid, formaldehyde, methanol, methane) that are the building blocks of the fuel and chemical industry. Unfortunately, the high thermodynamic stability of CO_2_ makes the conversion process difficult. Converting CO_2_ (a covalently bonded linear molecule) into fuel requires catalysts of high activity to add an electron (Figure 25). This is a challenging transformation as CO_2_^−^ has a bent geometry, and the activation energy is high [70]. In [69], it was demonstrated that the N_4_Mg_6_M (M = Li, Na, K) superalkalis can reduce CO_2_ and form superalkali/CO_2_ complexes in which CO_2_ is negatively charged.

**Figure 26:**
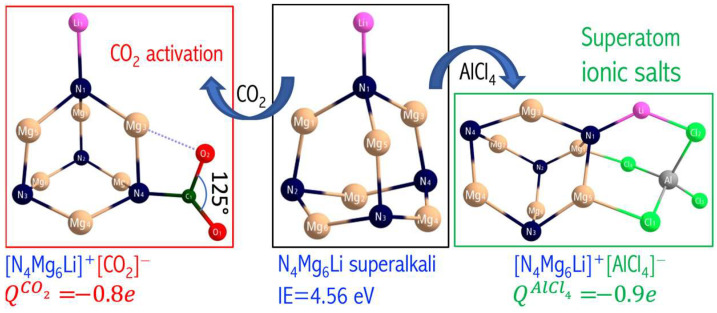
CO_2_ activation and ionic salt formation. Charge flow (Q) between superalkali and CO_2_/AlCl_4_. The plot is based on data from ref. [69]. Copyright 2020 AIP Publishing.

Since my goal was to design superatoms for chemical applications, the most important part of [69] was the assessment of the practicality and stability of superalkali/CO_2_ systems. The possibility of reducing carbon dioxide by N_4_Mg_6_M superalkalis was studied. Based on the ab initio calculations, it was demonstrated that N_4_Mg_6_M/CO_2_ systems represent two interacting ionic fragments (i.e., N_4_Mg_6_M^+^ and CO_2_^−^, Figure 26). In superalkali/CO_2_ complexes, the O–C–O bond angle was smaller (by 9%) than that in the isolated CO_2_^−^ anion and the amount of charge transfer was equal to 0.8 *e*. The charge transfer made possible by the low ionization energy of the superalkali seems to play a role in carbon dioxide activation, with a smaller ionization energy promoting the transfer of an electron to CO_2_. The bonding interaction between carbon and the electronegative atoms of the reductant also bends the linear CO_2_ structure. The above observations and their interpretations allowed us to consider N_4_Mg_6_M superalkalis as strong reducing agents.

Superalkalis can serve as building blocks of cluster-assembled materials or catalysts for CO_2_ activation. I indicated the superatom compounds that can be formed to serve as building blocks for cluster-assembled materials (Figure 26). I confirmed their structural stability. The high stability and donor-acceptor nature of the superalkali-superhalogen salts motivated me to design and evaluate a crystal structure of cluster-assembled superatomic compounds [71].

### 3.10. Molecular Crystals vs. Superatomic Lattice

**Figure 27:**
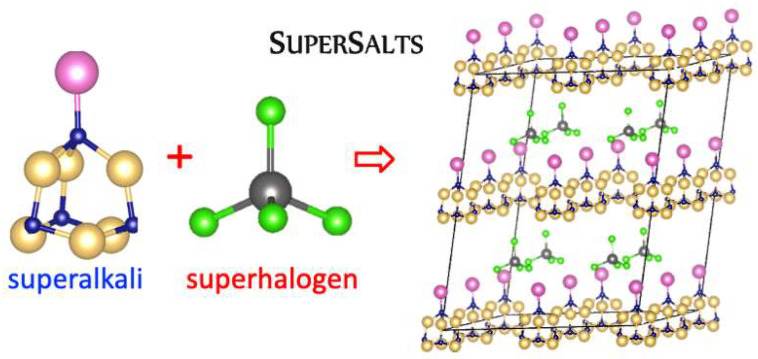
Binary [superalkali][superhalogen] salts.

In [69], I demonstrated that the designed N_4_Mg_6_M (M = Li, Na, K) superalkali systems form stable and strongly bound ionic compounds with superhalogens (AlCl_4_ and AlF_4_, Figure 26). The resulting [superalkali]^+^[superhalogen]^−^ supersalts reveal large cohesive energy (130–201 kcal/mol [69]), which increases with the increase in the ionization energy of superalkali. Hence, it is expected that [N_4_Mg_6_Li][AlCl_4_] systems might form stable solids [69]. Indeed, using a first-principles approach, we demonstrated that binary assemblies of superatomic compounds can be created through charge transfer between non-charged molecular clusters and utilizing intercluster electrostatic attraction as a driving force for co-assembly [71]. We combined pairs of complementary molecular clusters in which one cluster is electron-accepting (superhalogen) and the other is electron-donating (superalkali), Figure 27.

**Figure 28:**
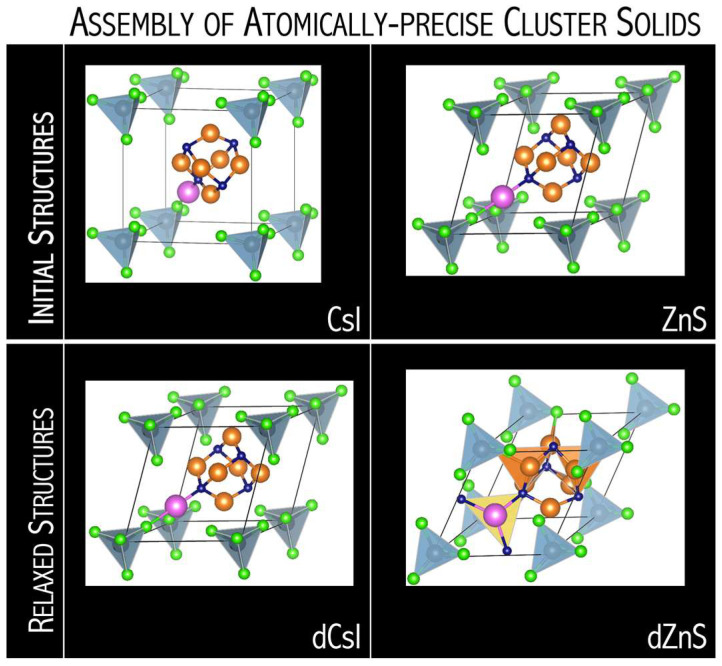
Initial and PBEsol relaxed structures of (N_4_Mg_6_Na)^+^(AlCl_4_)^−^ binary solids. Bulk-body-centred cubic (CsI), zinc blende (ZnS), and corresponding relaxed (deformed) structures dCsI and dZnS, respectively. Adapted with permission from ref. [71]. Copyright 2022 Royal Society of Chemistry.

Analysis of the first-principles results indicates that the [N_4_Mg_6_M]^+^[AlCl_4_]^−^ and [N_4_Mg_6_M]^+^[AlF_4_]^−^revealed a nanoscale atom nature by crystallizing in a similar packing as disordered zinc blende (dZnS, Figure 28), as well as also by resembling the empirical oxidation states of their atomic cation and anion analogs [71]. The N_4_Mg_6_M–AlX_4_ superatomic systems can also form molecular clusters where clusters are held to one another by electrostatic interactions (dCsI in Figure 28). The DFT results emphasize how the structure of superatomic solids can be tuned upon single-atom substitution. The competition between superhalogen size (*d*^SH^) and superalkali diameter (*d*^SA^) is a key factor for predicting the stability and crystalline form of superalkali-superhalogen supersalts. The N_4_Mg_6_M superalkalis form solids when combined with superhalogen oxidizing agents whose *d*^SA^/*d*^SH^ ratio is within the 1.32–1.78 range [71]. A larger *d*^SA^/*d*^SH^ ratio results in the close-packed superatomic lattice, whereas a smaller *d*^SA^/*d*^SH^ ratio favors the formation of larger intermolecular distances in molecular crystals. The lowest *d*^SA^/*d*^SH^ ratio (of 1.32) was found for N_4_Mg_6_Li–AlCl_4_, which forms molecular solids for dCsI and dNaCl phases. In turn, N_4_Mg_6_Na–AlCl_4_ and N_4_Mg_6_K–AlCl_4_ with larger *d*^SA^/*d*^SH^ ratios (1.44 and 1.46, respectively) favors close-packed superatomic phases forming [71].

The [superalkali][superhalogen] solids reveal the electronic structure and the band gap tunability. The ground states of N_4_Mg_6_Li–AlCl_4_, N_4_Mg_6_K–AlCl_4_, and N_4_Mg_6_K-AlF_4_ solids are semiconductors with indirect band gap (0.122, 0.151, and 0.103 eV) [71]. In turn, in N_4_Mg_6_Na–AlCl_4_, N_4_Mg_6_Li–AlF_4_, and N_4_Mg_6_Na–AlF_4_ solids, the Fermi level cuts across the valence band, revealing a metallic nature. The substitution of Cl by F reduces the band gap from 0.122 eV (N_4_Mg_6_Li–AlCl_4_) to 0.000 eV (N_4_Mg_6_Li–AlF_4_) and from 0.151 eV (N_4_Mg_6_K–AlCl_4_) to 0.103 eV (N_4_Mg_6_K–AlF_4_) [71]. The band gap changes on halogen atom substitution are accompanied by valence band dispersion increase and are influenced by electronegativity (larger electron affinity). Based on our calculations, the electronic structures of the superalkali-superhalogen solids can be modified via alkali metal/halogen substitution, which provides GAP tunability. Overall, the first-principles results described in [71] demonstrate how modification of the superatoms allows for tuning of the superstructure and electronic configuration and can be used to establish desirable functional materials.

The analysis of the first-principles computations proved the tunability of electronic and crystal structures of supersalts upon individual superatom modification. The DFT computations not only conform to the ability of superatoms to serve as building blocks of chemical materials but also allow us to predict the influence of superatomic structure modification on the electronic structure of the resulting materials. The new knowledge obtained within [71] can be used to form functional materials with desirable properties. In this way, superatoms can replace elements of the periodic table, especially those with increasing scarcity. Nowadays, we risk seeing many of the natural elements that make up the world around us run out due to limited supplies, their location in conflict areas, or the incapacity to fully recycle them. Substitutes for scarce elements can be obtained by replacing them with superatoms. Superatoms can be made of combinations of plentiful supply atoms. Thus, the ability to design superatoms offers a new concept for materials design and the opportunity for their electronic structure to be tuned through minor, targeted modifications of their composition that take advantage of their predictable electronic structure.

## 4. Summary—The Elements of Scientific Novelty

The results obtained and described in this feature paper demonstrate the chemical potential of superatoms. The most important, in terms of cognitive and practical, scientific achievements include:

▪ The successful determination of the structure features that strongly influence the electron affinity of superhalogens and the development of a mathematical model for the VDE prediction. The developed QSPR model predicts the oxidizing ability of superhalogen species based on topology (structure optimization with extensive quantum mechanical calculations is not required). The model can be used for quickly designing new superhalogens with a desired electron affinity.
▪ The design of alternative superhalogen anions with inert noble gas atoms playing the role of central atoms and the influence of a number of electronegative ligands on their electronic stability analysis.
▪ Finding the relationship between the electronegativity of ligands and the thermodynamic and electronic stability of superhalogen anions. The electron affinity of ligands and collective effects play a crucial role in superhalogen systems and determine their electronic stability.
▪ The design of polynuclear superhalogens consists of mixed ligands (F, O) and the description of the influence of introducing mixed ligands on the HOMO nature and electronic stability of the resulting system.
▪ Examination of the oxidizing potential of mono- and polynuclear superhalogens. Superhalogens can effectively ionize moderately reactive molecules (such as fullerene C_60_ or metal oxides), and this ability increases with the electron affinity of superhalogen increase.
▪ The development of the recommendation on utilizing polynuclear superhalogen anions as electrolytes in lithium-ion batteries with desirable physicochemical properties (in the frame of the “safe-by-design” idea).
▪ Utilizing superalkali cations to design novel superalkalium-PbBr_3_ perovskites with large stability and excellent electronic features, which can be useful in optoelectronic devices such as solar cells.
▪ The design of new superalkali systems demonstrates their strong reducing ability, enabling even the activation of chemically inert carbon dioxide (CO_2_).
▪ The design and optimization of binary crystal structures made of superalkali cations and superhalogen anions. The designed supersalt are semiconductors ([N_4_Mg_6_Li]^+^[AlCl_4_]^−^, [N_4_Mg_6_K]^+^[AlCl_4_]^−^, [N_4_Mg_6_K]^+^[AlF_4_]^−^) or have a metallic nature ([N_4_Mg_6_Na]^+^[AlCl_4_]^−^, [N_4_Mg_6_Li]^+^[AlF_4_]^−^, [N_4_Mg_6_Na]^+^[AlF_4_]^−^). The electronic structure of supersalts can be easily tuned by a replacement, even a single atom within an individual superatom, which can be used to create functional materials with desirable physicochemical properties.

## 5. Research Methods

The designed superatoms need to be the global minimum in the potential energy surface to serve as the target for experimental synthesis [26,72]. The molecular structure search for neutral and ionic superatoms was based on ab initio calculations and both manual and the Calypso structure searching methods [73]. The Calypso structure prediction method has been successfully applied to the prediction of reliable structures for various compounds [74]. In the next step, the lowest-energy candidate structures of the global minimum were further improved by performing geometric optimization using the second-order Møller-Plesset (MP2) perturbational method with the Pople split-valence basis sets of triple zeta quality, 6-311+G(d), as implemented in the Gaussian 16 package [75]. The vibrational frequency calculations were performed at the same level of theory to check the nature of the stationary points. The coupled-cluster method with single, double, and non-iterative triple excitations, CCSD(T), with the 6-311+G(3df) basis, was used to calculate the final energies of the species at their equilibrium geometries calculated with the MP2/6-311+G(d) approach.

In the case of the larger systems (such as Co_6_Se_8_(P(C_2_H_5_)_3_)_6_, Cr_6_Te_8_(P(C_2_H_5_)_3_)_6_, Ni_9_Te_6_(P(C_2_H_5_)_3_)_8_, and C_60_), where MP2 calculations are too expensive for the computer resources that we have at hand, density functional theory was applied. We will apply three exchange–correlation functionals, the Perdew–Burke–Ernzerhof (PBE) functional [76], PBE0, and HSE06 hybrid functionals [77]. For the PBE functional, we included the dispersion terms by Grimme’s D3 dispersion model with Becke–Johnson damping (PBE-D3) [78].

The oxidizing (superhalogens) and reducing (superalkalis) abilities of designed molecular clusters were evaluated from electron affinity and ionization energy, respectively. The adiabatic electron affinity (AEA) indicates the ability of the species to attach an extra electron to form a stable system. We calculated the AEA of superhalogen by subtracting the total electronic energies of the neutral (E_neu_) and anionic species (E_an_) at each equilibrium geometry (r_e,0_ and r_e,-_, respectively; Equation (6)). The vertical electron detachment energy (VDE) is defined as the minimum energy needed to detach an electron from the anion in its ground state and was estimated as the electronic energy differences between the neutral and anion, both at the equilibrium geometry of the anionic system (r_e,-_; Equation (7)). The adiabatic ionization energy (AIE) indicates the ability of the neutral system to detach an electron to form a stable cation. The AIE was estimated by subtracting the total electronic energies of the cation (E_cat_) and neutral species (E_neu_) at each equilibrium geometry (r_e,+_ and r_e,0_, respectively; Equation (8)). The vertical ionization energy (VIE) is defined as the minimum energy needed to detach an electron from the non-charged system in its ground state and was estimated as the electronic energy differences between the cation and neutral, both at the equilibrium geometry of the neutral system (r_e,0_; Equation (9)).

AEA = E_neu_(r_e,0_) − E_an_(r_e,-_) (6)

VDE = E_neu_(r_e,-_) − E_an_(r_e,-_) (7)

AIE = E_cat_(r_e,+_) − E_neu_(r_e,0_) (8)

VIE = E_neu_(r_e,0_) − E_an_(r_e,0_) (9)

The adiabatic ionization energies (AIEs) and vertical ionization energies (VIEs) were calculated using the CCSD(T)/6-311+G(3df)//MP2/6-311+G(d) approach and comprising zero-point energy corrections.

The intramolecular interaction energies (E_int_) of the superatom/**Y** systems were obtained at the CCSD(T)/6–311+G(d) level of theory. To eliminate the basis set superposition error (BSSE) effect, we used the counterpoise procedure, in which the same basis set (E_superatom/**Y**_) was applied for the subunit energy (E_superatom_ and E**_Y_**) computations as for the complex energy (E_superatom/**Y**_) estimation; Equation (10).

E_int_ = E_superatom/**Y**_(X_superatom/**Y**_) − E_superatom_(X_superatom/**Y**_) − E**_Y_**(X_superatom/**Y**)_ (10)

We used a first-principles calculation to design [superalkali][superhalogen] lattice and superalkali-based perovskite structures. We performed calculations with the Vienna ab initio simulation package (Vasp) [79,80,81,82]. We performed a full structure relaxation without symmetry restrictions using the Perdew–Burke–Ernzerhof revised for solids functional (PBEsol). We chose this function as it was shown to exhibit excellent accuracy in predicting the structural parameters of molecular solids [60,83]. Band structures and density of states (DOS) were performed with PBE-D3 functional incorporated dispersion correction by Grimme et al. with Becke–Johnson damping [78]. We examined the electronic structure of the studied clusters based on the band structure and density of states analysis obtained at the HSE06-D3 level incorporated dispersion correction by Grimme et al. with Becke–Johnson damping [78].

Cohesive energy (E_coh_) was calculated as the difference between the total energy of the [superalkali][superhalogen] bulk and the individually relaxed counterparts (i.e., superhalogen and superalkali), Equation (11).

E_coh_ = E_superalkali/superhalogen_ − E_superalkali_ − E_superhalogen_ (11)

## 6. Future Research Topics

Superatoms are a rapidly growing topic of interest in materials science, bringing the chemistry of atomically precise clusters together with physics. Materials composed of superatoms, the so-called cluster-assembled solids, hold the promise of atomic precision, high tunability, and robust architectures. Superalkalis are a class of superatoms that have extremely low ionization energies and might serve as reducing agents. It is desirable to design new superalkalis and examine their electronic features to meet their chemical potential as building blocks for materials. By using the superalkalis as a building block, we can achieve the desirable functional characteristics of atom-based materials.

Superalkalis can be explored as catalysts for N_2_ activation and conversion into ammonia. The Haber-Bosch process is the predominant source of the world’s ammonia (NH_3_) production (175 million metric tons) and represents more than 90% of the annual production. Despite significant efforts in optimizing the process, it still consumes from 1 to 2% of energy worldwide because the production process requires high temperatures (at 573–873 K) and pressures (at 100–360 atm), currently producing more than 1.6% of global carbon dioxide emissions. Therefore, developing efficient catalysts that convert N_2_ into ammonia is vital to reduce the growing energy crisis and global warming. The high stability of N_2_ makes the conversion difficult. This is a challenging transformation as N_2_ has a N≡N triple bond, and the activation energy is high (941 kJ/mol). In light of the above challenges, my future research directions focus on the study of existing and designing new superalkalis as elements of unique materials with useful physical and chemical properties in terms of applications (such as N_2_ conversion into ammonia).

My next research direction aims to answer the question as to whether the active CO_2_ molecule (activated upon interaction with the superalkali system) can be further transformed into other valuable chemicals by interacting with other chemical species. A good catalyst for CO_2_ conversion needs to reduce CO_2_ but should not bind the activated CO_2_^−^ too strongly. The ability of the activated CO_2_ to transform is crucial for CO_2_ conversion into value-added chemicals (such as CO, CH_4_, or C_2_H_4_). Promising processes might be the cycloaddition of CO_2_ with propylene oxide or radical reactions with small molecules (i.e., H_2_, CH_4_). Also, the question is whether the superalkali systems might capture visible light to drive catalytic reactions and achieve high conversion efficiency at low temperatures. This would indicate the great potential of superalkalis as efficient photocatalysts for converting CO_2_ to high-value chemicals.

## Data Availability

The data presented in this study are available in [36,43,45,46,49,50,51,56,58,61,69,71].
